# Salt-induced expression of intracellular vesicle trafficking genes, *CaRab*-GTP, and their association with Na^+^ accumulation in leaves of chickpea (*Cicer arietinum* L.)

**DOI:** 10.1186/s12870-020-02331-5

**Published:** 2020-10-14

**Authors:** Crystal Sweetman, Gulmira Khassanova, Troy K. Miller, Nicholas J. Booth, Akhylbek Kurishbayev, Satyvaldy Jatayev, Narendra K. Gupta, Peter Langridge, Colin L.D. Jenkins, Kathleen L. Soole, David A. Day, Yuri Shavrukov

**Affiliations:** 1 College of Science and Engineering, Biological Sciences, Flinders University, Adelaide, Australia; 2 Faculty of Agronomy, S. Seifullin Kazakh AgroTechnical University, Nur-Sultan, Kazakhstan; 3 College of Agriculture, SKN Agriculture University, Jobner, Rajasthan India; 4 Wheat Initiative, Julius-Kühn-Institute, Berlin, Germany; 5 School of Life Science, AgriBio, LaTrobe University, Melbourne, Australia

**Keywords:** Chickpea, Gene expression, *Rab*-GTP genes, Salt stress, Salinity, Vesicle trafficking

## Abstract

**Background:**

Chickpea is an important legume and is moderately tolerant to salinity stress during the growing season. However, the level and mechanisms for salinity tolerance can vary among accessions and cultivars. A large family of *CaRab*-GTP genes, previously identified in chickpea, is homologous to intracellular vesicle trafficking superfamily genes that play essential roles in response to salinity stress in plants.

**Results:**

To determine which of the gene family members are involved in the chickpea salt response, plants from six selected chickpea accessions (Genesis 836, Hattrick, ICC12726, Rupali, Slasher and Yubileiny) were exposed to salinity stress and expression profiles resolved for the major *CaRab*-GTP gene clades after 5, 9 and 15 days of salt exposure. Gene clade expression profiles (using degenerate primers targeting all members of each clade) were tested for their relationship to salinity tolerance measures, namely plant biomass and Na^+^ accumulation. Transcripts representing 11 out of the 13 *CaRab* clades could be detected by RT-PCR, but only six (*CaRabA2*, *−B*, *−C*, *−D*, *−E* and −*H*) could be quantified using qRT-PCR due to low expression levels or poor amplification efficiency of the degenerate primers for clades containing several gene members. Expression profiles of three gene clades, *CaRabB*, *−D* and *−E*, were very similar across all six chickpea accessions, showing a strongly coordinated network. Salt-induced enhancement of *CaRabA2* expression at 15 days showed a very strong positive correlation (R^2^ = 0.905) with Na^+^ accumulation in leaves. However, salinity tolerance estimated as relative plant biomass production compared to controls, did not correlate with Na^+^ accumulation in leaves, nor with expression profiles of any of the investigated *CaRab*-GTP genes.

**Conclusion:**

A coordinated network of *CaRab-GTP* genes, which are likely involved in intracellular trafficking, are important for the salinity stress response of chickpea plants.

## Background

The *Rab*-GTP gene family encodes small guanidine triphosphatase (GTP)-binding proteins, which exist alongside other groups of similar genes, *Arf*, *Ran, Rho* and *Ras*. *Rab*-GTPs are ubiquitous in eukaryotes and are involved in vesicle trafficking within cells. Rab-GTP proteins are present on all intracellular membranes including the entire endomembrane system (endoplasmic reticulum, Golgi apparatus, lysosome and endosome), as well as on nuclear, mitochondrial, vacuolar and plasma membranes [1–5]. Some *Rab*-GTPs are also localised on plant chloroplast membranes [6]. Rab-GTPs perform diverse functions including exo- and endocytosis, membrane differentiation, organelle development, fission and motility, cell division, signalling, and organogenesis [7, 8]. GTP hydrolysis regulates the transition between active (GTP-bound) and inactive (GDP-bound) forms [9]. GDP/GTP Exchange Proteins or Factors (GEPs or GEFs), including Transport Protein Particle II (TRAPPII) and GDP Dissociation Inhibitors (GDIs), enhance and inhibit the transition process between the two forms, respectively [10]. GDIs retain Rab-GTPs in their GDP-bound form, which leads to dissociation of the Rab-GTP from its membrane and this, along with Rab Escort Proteins (REPs) and prenylation of Rab-GTPs, enables recycling and re-targeting of the Rab-GTP to new membranes for multiple rounds of vesicle transport [10–16].

Intracellular trafficking processes regulated by Rab-GTP proteins are vitally important for plant development, including pollen tube growth [17], fruit ripening [18], root and nodule development in legumes [19–21], and hypocotyl growth [22], and are involved in the plant’s response to various biotic [23–25] and abiotic stresses [26, 27]. Plant *Rab*-GTP genes can be classified into eight out of nine possible clades based on their predicted protein structures [28], designated as clades A-H [29–31].

RabA (Rab11) members mediate transport between the *trans*-Golgi network and the plasma membrane. In *Arabidopsis thaliana*, the RabA2a protein was reported as an interacting partner of TRAPPII [10]. *RabA*-GTP genes are directly involved in plant responses to NaCl and are required for salt stress tolerance. Four major *AtRabA1* members were knocked out in the *A. thaliana* mutant line *rabA1b*, resulting in hypersensitivity to salinity [32], while transgenic rice overexpressing *OsRabA* (also known as *OsRab11*) displayed higher salinity tolerance compared to wild-type plants [33]. *RabB* (*Rab2*) members have also been shown to be responsive to abiotic stresses. *SsRabB* (also *SsRab2*) transcript levels in the desiccation-tolerant grass *Sporobolus stapfianus* increased in response to dehydration and then decreased after rehydration [34], while *LfRab* from the Easter lily, *Lilium formolongi* was induced by both drought and salt stress [35]. *RabC* (*Rab18*), *RabD* (*Rab1*) and *RabE* (*Rab8*) genes have been less studied in plant stress experiments. However, in our previous study we determined that chickpea *CaRabC* genes were highly expressed in response to salinity and rapid dehydration but down-regulated by drought [36]. In poplar, the overexpression of a constitutively activated *PtRabE1b* enhanced growth in the presence of salt [37].

*RabF* (*Rab5*) and *RabG* (*Rab7)* mainly regulate vacuolar trafficking to and from the pre-vacuolar compartments [27, 38–41]. The encoded proteins interact with tethering complexes, including ‘Class C core vacuole/endosome tethering’ (CORVET) and ‘Homotypic fusion and protein sorting’ (HOPS) complexes, which mediate between trafficking vesicles and target membranes [42]. The effect of salinity on endocytosis and intracellular trafficking has been reviewed earlier [43, 44]. In *Arabidopsis*, *RabF* genes are present in early endosomes and involved in trafficking from the Golgi to the pre-vacuolar compartments [45–47]. *AtRabF* (*AtRab5*) encodes the ARA6 protein, which has a functional role in salt stress [5]. In *Mesembryanthemum crystallinum*, transcript levels of *McRabF* (also called *McRab5b*) increased over 3 days of salt stress, but were not responsive to leaf wilting and osmotic stress [48]. *RabG* is also a key-player during abiotic stress across diverse species. Transcript levels of *OsRabG* (*OsRab7*) and *AlRabG* (*AlRab7*) were up-regulated by dehydration and salinity in rice and in the halophytic grass *Aeluropus lagopoides*, respectively [49, 50]. Overexpression of endogenous *RabG* genes led to enhanced salinity tolerance in *Arabidopsis* (*AtRabG* [51]), rice (*OsRabG* [52]) and peanut (*AhRabG* [53];), while expressing *RabG* genes from the abiotic stress-tolerant plants *Pennisetum glaucum* (a drought-tolerant grain crop) and *Prosopsis juliflora* (a Fabaceae tree) in tobacco also conferred salinity tolerance (*PgRabG/PgRab7* and *PjRabG*/*PjRab7* [54, 55]). *RabH* genes have not been studied as much, but two *Arabidopsis* genes, *AtRabH1b* and *AtRabH1c*, have been shown to localise to the Golgi in *Arabidopsis* and tobacco plants, and the former also to an undetermined compartment [56]. *AtRabH1b* was also shown recently to modulate the trafficking of cellulose synthase complexes between endomembrane compartments and the plasma membrane [22].

As described above, there are many examples of salt-induced expression of *Rab*-GTP genes and improved salinity tolerance in plants over-producing Rab-GTP proteins. Transgenic *Arabidopsis*, rice and tobacco plants overexpressing *Rab*-GTPs also accumulate more sodium in plant tissues during salt stress [51, 52, 55], and the rice overexpression lines have increased numbers of vesicles at the root tip. This suggests that the improved salinity tolerance was not due to sodium exclusion, but rather sequestration. In addition to increased rates of endocytosis and Na^+^ accumulation in the vacuoles of roots and shoots, the *Arabidopsis* overexpression lines also demonstrated decreased levels of Reactive Oxygen Species (ROS), possibly due to decreased NADPH oxidase activity [51].

The *Rab*-GTPs of chickpea (*Cicer arietinum*) have received little attention, but recent accessibility of the genome sequence and transcriptomic databases has made it possible to identify 54 *CaRab* genes [36], representing each of the eight plant *Rab*-GTP clades: 24 *CaRabA* genes, 3 *CaRabB*, 5 *CaRabC*, 4 *CaRabD*, 5 *CaRabE*, 2 *CaRabF*, 7 *CaRabG* and 4 *CaRabH* genes. The large *CaRabA* gene clade was further divided into sub-clades (with 8 *CaRabA1*, 4 *CaRabA2*, 2 *CaRabA3*, 5 *CaRabA4*, 3 *CaRabA5* and 2 *CaRabA6* genes). Khassanova et al. [36] also reported that *CaRabC* genes are highly regulated by abiotic stresses, particularly salinity and desiccation. The aim of the present study is to determine which other *CaRab* genes are expressed in *C. arietinum* and to identify key-players in the response to salinity stress. Six chickpea accessions were chosen based on their commercial use and salinity tolerance [36, 57, 58].

## Results

### Growth of chickpea plants in the presence of salt

**Fig. 1:**
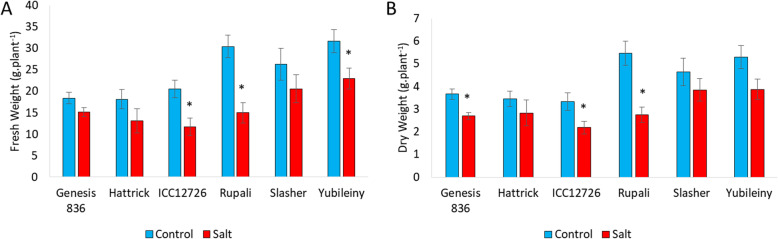
Effect of salt exposure on plant biomass. **(a)** Fresh weights and **(b)** dry weights of whole chickpea shoots grown in the presence or absence of 90 mM NaCl. Plants were grown for 1 month before salt application, and then exposed to salinity for 1 month before harvesting. * *p* < 0.05 based on unpaired *t*-tests between control and salt groups for individual genotypes (*n* = 8 ± S.E.M.)

All six chickpea germplasm accessions showed some form of growth impairment in the presence of NaCl, demonstrated by decreased fresh weight (FW) and dry weight (DW) (Fig. 1), and visual symptoms including chlorosis, browning and senescence (Additional file 1, Fig. S1-S2).

After 1 month of salt exposure, plants of cv. Rupali were the most severely affected, retaining only 50% of both FW and DW relative to plants grown in control conditions. Two accessions, ICC12726 and Yubileiny, were judged as moderately sensitive to salinity, while Genesis 836 and Slasher remained relatively unaffected and salt tolerant. Plants of Hattrick did not show impaired weights but were visually affected with clear symptoms of sodium toxicity (Additional file 1, Fig. S2).

### Accumulation of Na^+^ and K^+^ in leaves of chickpea plants

**Fig. 2:**
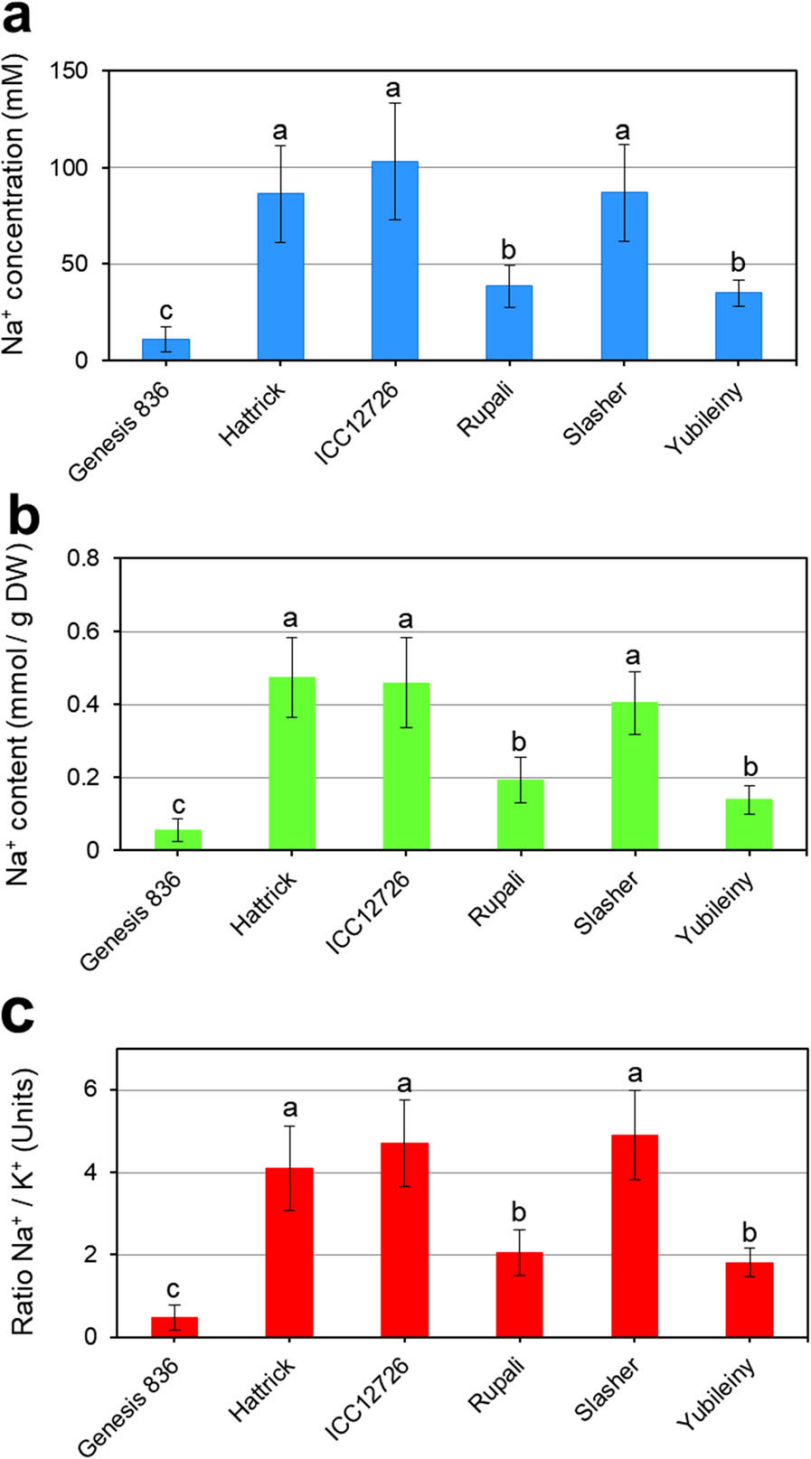
Accumulation of Na^+^ and K^+^ in leaves of salt-treated chickpea plants. **(a)** Based on sap, or leaf water content, **(b)** based on leaf dry weight, and **(c)** the ratio of Na^+^/K^+^ calculated from the same samples. Significant differences are indicated by different letters (p < 0.05), calculated using ANOVA with post-hoc Tukey tests (n = 8 ± S.E.M.)

Levels of Na^+^ and K^+^ were measured in the youngest fully developed leaves from plants sampled 1 month after NaCl application. There was considerable variability between replicate plants, which may explain some of the variability seen in other measurements. Despite this, clear trends are evident, with up to 10-fold differences in Na^+^ concentration in the sap of the chickpea accessions with the highest and lowest level of Na^+^. Plants of Genesis 836 contained the lowest concentration and content of Na^+^, 10 mM (Fig. 2a) and 0.05 mmol/g DW (Fig. 2b), and the Na^+^/K^+^ ratio (0.5) was low (Fig. 2c). In contrast, plants of Hattrick, ICC12726 and Slasher all had very high Na^+^ levels, up to 100 mM and approximately 0.5 mmol/g DW, and Na^+^/K^+^ ratios of up to 4.5. Rupali and Yubileiny had low-to-moderate Na^+^ levels (about 40 mM of Na^+^ concentration in sap and 0.15–0.2 mmol Na^+^ content /g DW), and moderate Na^+^/K^+^ ratios (about 2.0) (Fig. 2). Because there were no significant differences between the two calculation methods for Na^+^ accumulation (in the sap and in dried leaf samples), we used the Na^+^/K^+^ ratio for further correlation analysis.

### Analysis of *CaRab* gene expression

**Fig. 3:**
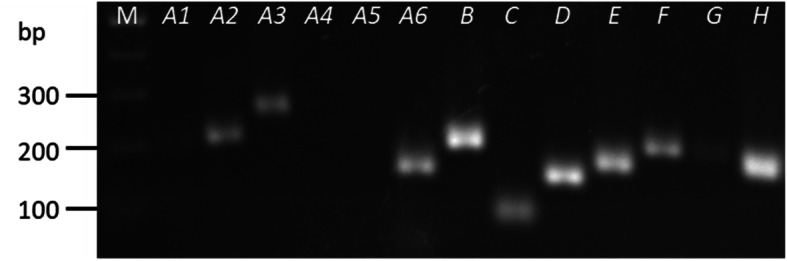
Products of semi-quantitative RT-PCR for 13 *CaRab* gene clades using a pooled cDNA samples from all six chickpea accessions, including control and salt stress (90 mM NaCl) samples. 100 bp DNA ladder (Bioline) for amplicon size estimation. Expected sizes of amplified product (bp) of *CaRab* genes: *CaRab-A1*, 193; *−A2*, 210; *−A3*, 150; *−A4*, 145; *−A5*, 161; *−A6*, 159; *−B*, 194; *−C*, 88; *−D*, 146; *−E*, 165; *−F*, 199; *−G*, 190; and *−H*, 197

Previous work has established a classification system for the *CaRab*-GTP gene family [36]. As there are at least 54 *CaRab* genes, degenerate primers were designed to target each major clade or sub-clade rather than individual genes, by targeting the regions of highest homology between clade members (Additional file 1, Fig. S3). Products of expected sizes were obtained for primer sets of all clades except for the closely related *CaRabA4* and *−A5* (Fig. 3). Primers for clades *CaRabA4* and *−A5* were highly degenerate. Primers with appropriate efficiency for qPCR were obtained for all remaining clades except *CaRabA3* and −*A6*. *CaRabA1, −F* and −*G* were below quantification limits and therefore could not be measured. *CaRabA2*, −*B*, −*C*, −*D*, −*E* and −*H* amplified well with good primer efficiency for qPCR.

**Fig. 4:**
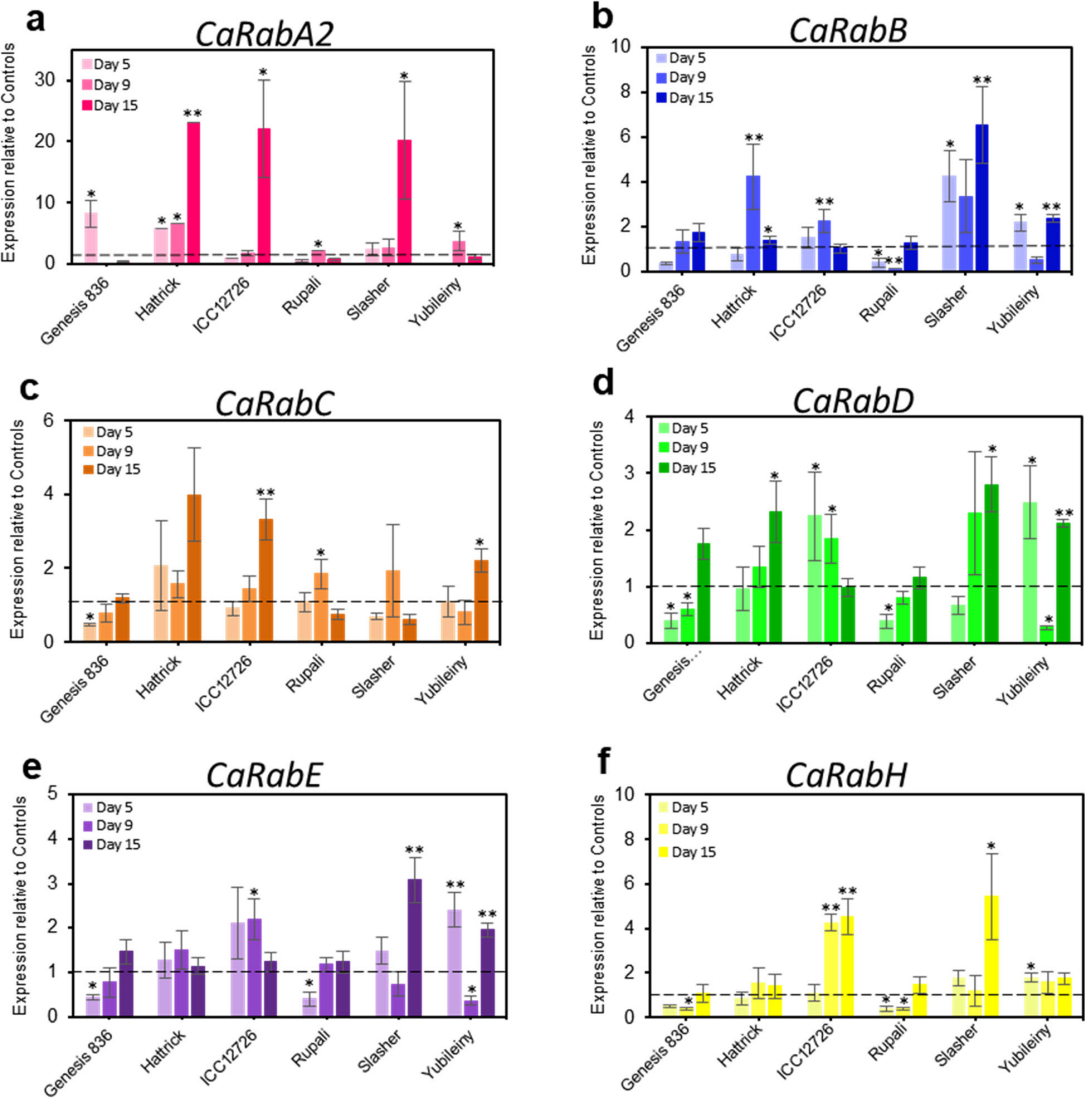
Quantitative RT-PCR analysis of *CaRab* genes in six chickpea accessions exposed to salt stress (90 mM NaCl). Effect of salt treatment on transcript levels of genes: **(a)** *CaRabA2*; **(b)** *CaRabB*; **(c)** *CaRabC*; **(d)** *CaRabD*; **(e)** *CaRabE*; and **(f)** *CaRabH*. Samples were collected at three time-points, based on the number of days after the initial salt addition. Gene expression data were normalised using average units relative to transcript levels of two Reference genes, *CaHsp90*, Heat shock protein (GR406804) and *CaEf1α*, Elongation factor Elongation factor 1-alpha (AJ004960). Bars represent means relative to control plants across all time-points, which are set as 1 unit and indicated by dash-lines. *p < 0.05, **p < 0.01 based on in two-way ANOVA with post-hoc Tukey test between control and salt groups for individual genotypes (*n* = 4–8 ± S.E.M.)

Transcripts in the leaves of the six chickpea genotypes grown with or without salt stress were quantified using qRT-PCR (Fig. 4). Transcript levels of the *CaRab* genes from clades *A2*, *B*, *C*, *D*, *E* and *H* were measured at three time-points: ‘early’ (Day 5; collected 5 days since the first salt application), ‘middle’ (Day 9) and ‘late’ (Day 15). Due to the transient nature of *Rab* gene regulation, early sampling time-points were necessary to obtain good expression profiles of each gene.

Expression of the various *CaRab* gene clade members differed between genotypes and in response to salt. However, *CaRabA2* expression was significantly and strongly enhanced (about 20-fold) at the late time-point in Hattrick, ICC12726 and Slasher. Positive, but less pronounced, changes in the expression of *CaRabA2* were also observed in the other cultivars and at earlier time points under salt stress (Fig. 4a).

Expression of members of clades, *CaRabB*, *−D* and *−E* was similar across most of the chickpea accessions and time-points (Fig. 4b, d and e). For example, in Genesis 836 and Rupali, expression of *CaRabB*, −*D* and −*E* was down regulated at early time-points, but increased at the latest time-point, while Yubileiny showed a double peak in expression at the early and late time-points but decreased significantly at the middle time-point. Hattrick, ICC12726 and Slasher showed more variable, but nontheless significant, up-regulation of the *CaRabB*, −*D* and −*E* genes at one or more time-points. Pearson’s correlation analysis confirmed high similarity (R^2^ = 0.640–0.754, *p* < 0.01) in the expression trends of these three genes.

With the exception of Genesis 836, expression of *CaRabC* was similar across all chickpea accessions studied, showing significant, but variable, increases at different time-points, notably 2–4-fold at the late time-point for Hattrick, ICC12726 and Yubileiny (Fig. 4c). Very different expression profiles were found for *CaRabH* in the accessions, with expression down-regulated in Genesis 836 and Rupali, as observed also for *CaRabB*, −*D* and −*E*, while Yubileiny, ICC12726 and Slasher showed increased expression at early, middle-late and late time-points (Fig. 4f).

### Correlation analysis of Na^+^ accumulation and *CaRab* gene expression

**Fig. 5:**
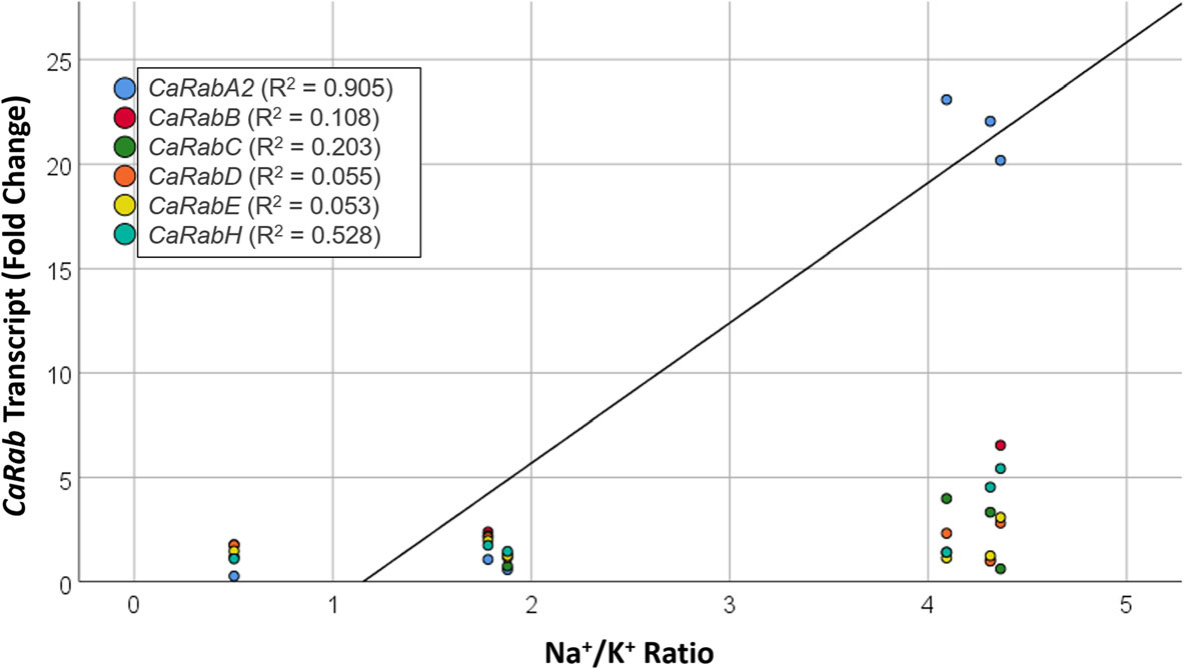
Linear regression of the six *CaRab* gene expression profiles, expressed as fold-differences compared to Controls, and Na^+^ accumulation in leaf samples, expressed as a Na^+^/K^+^ ratio. IBM SPSS Statistical software was used for Pearson’s correlation analysis (IBM SPSS, Statistics Desktop 25.0.0.0). Line of best fit is shown only for *CaRabA2*, for clarity

Linear regression analyses were used to further explore the relationship between *CaRab* gene expression, Na^+^ accumulation and salinity tolerance across the six chickpea cultivars. Expression of all genes and between individual genes were compared to biomass and Na^+^/K^+^ data. A very strong correlation (R^2^ = 0.905, *p* < 0.01) was observed between the Na^+^/K^+^ ratio in leaves and the expression profile of *CaRabA2*, after 15 days of plant exposure to salt stress (Fig. 5). No significant correlations were observed between Na^+^/K^+^ ratio and any other gene or time-point. Similarly, no significant correlations were observed between relative biomass and any gene at any time-point.

## Discussion

Previous studies with *Arabidopsis* have shown that Rab-GTP polypeptides are found on almost all intracellular membranes and appear to be involved in a coordinated network for intracellular trafficking of important substances. Some are also regulated by the Transport Protein Particle II (TRAPPII) system, which facilitates exchange between GTP and GDP and thereby can activate or repress Rab-GTP [10, 59]. The models generated for this network are based on evidence from *Arabidopsis* tissues grown under non-stressed conditions [10, 40, 60]. In the latest model [10], RabD protein is associated with the Rough Endoplasmic Reticulum (RER), while RabB and RabA are localized in the Golgi and *trans*-Golgi network/Early endosome (TGN/EE), respectively. Members of the RabD and RabA2 clades are regulated by TRAPPII. Secretory vesicles (SV) produced by the TGN/EE are transported along microtubules, directed by RabH, and then dock at the plasma membrane, controlled by RabE. Clathrin-coated vesicles (CCV) are also produced by TGN/EE, but these vesicles are transported directly to vacuoles under the control of RabF and RabG.

In the present study, transcripts representing 11 of the 13 *CaRab* gene clades were detected, but only six could be quantified using qRT-PCR. Five of these clades (*CaRabA2*, *−B*, *−D*, *−E* and *−H*) contained genes with orthologs in *Arabidopsis* [10], with the exception of *CaRabC*. This is not surprising, as dicot species like *A. thaliana* and *C. arietinum* share very similar genes. *RabC*, which has received little attention in the plant literature, has been shown previously to respond to abiotic stress in chickpea [36]. Expression profiles of six *CaRab* gene clades were obtained for six chickpea accessions differing in salinity tolerance, defined as biomass of salt-treated plants relative to control plants after 1 month of exposure to 90 mM NaCl in soil.

While each accession had a genotype-specific pattern of *CaRab*-GTP gene expression, the expression of three gene clades, *CaRabB*, *−D* and *−E*, were similar and these genes may, therefore, act as part of a coordinated network regardless of growth conditions. The observed co-regulation of these three clades may reflect their involvement in vesicle trafficking from the Golgi apparatus and RER to the plasma membrane. While expression of individual members of this ‘trio’ of *CaRab*-GTP genes was strongly coordinated within each accession, their expression pattern was quite different across the six accessions. For example, their response to salinity in Genesis 836 was very different from that in Yubileiny. This may reflect the very different backgrounds of the chickpea accessions, resulting in genotype-specific responses, but may also reflect the multi-faceted response of plants to salt, which involves many other genes [61].

The expression of the *CaRabA2* gene clade showed a strong positive correlation with Na^+^ accumulation (Fig. 5). Three chickpea accessions, Hattrick, ICC12726 and Slasher accumulated high Na^+^ in leaves (Fig. 2), and *CaRabA2* expression was strongly enhanced (about 20-fold) in these cultivars after 15 days of NaCl exposure. In the other three accessions, Genesis 836, Rupali and Yubileiny, which had low Na^+^ accumulation, *CaRabA2* gene expression was not up-regulated (Fig. 4). The precise role of *CaRabA2* in Na^+^ accumulation in leaves has not yet been determined, but *RabA* members are generally thought to mediate transport between the *trans*-Golgi network and the plasma membrane [60]. Therefore, we can speculate that *CaRabA2* controls the formation of SV and/or CCV, encapsulating cytosolic Na^+^ and trafficking it to the plasma membrane, vacuole or both. It seems that up-regulation of *CaRabA2* expression occurs after accumulation of Na^+^ in the cytoplasm of leaf cells, taking up to 15 days for the maximum response. This presumably resulted in increased *CaRabA2* protein at the TNG/EE with a concomitant increase in the capacity for Na^+^ trafficking to other compartments of the cell.

Due to poor amplification and amplification efficiency of *CaRabF* and *CaRabG*, respectively, it is not possible to make conclusions from our experiments about the expression of these gene clades and their involvement in the response of chickpea plants to salt stress. CaRabF and CaRabG proteins localise to the pre-vacuole compartment and membrane and control trafficking to the vacuole [38, 39, 41]. They are also important in trafficking of Na^+^ ions, based on transgenic studies [5, 51]. Interestingly, in the presence of salt, the localisation of *Arabidopsis* RabF protein ARA6 shifts from the pre-vacuolar compartment to the plasma membrane [5], suggesting dual directionality of trafficking that is dependent on cellular environment. It is unknown whether this phenomenon occurs for other Rab-GTP proteins in plants.

Several *Rab*-GTP overexpression studies have noted that improved plant survival was accompanied by high Na^+^ accumulation [51, 52, 55], suggesting that sequestration is an effective means for salt tolerance and that Rab-GTP trafficking is important for Na^+^ compartmentalisation. However, Rab-GTPs have diverse functions and the specific mechanism for enhanced Na^+^ relocation may vary. For example, *Rab*-directed trafficking of Na^+^-containing vesicles can occur from the plasma membrane to the vacuole in *Arabidopsis* cell cultures [62]. In addition, Rab-GTPs are involved in fusion of endosomes, pre-vacuole compartments and vacuoles in yeast and mammalian cells [63, 64], which could increase storage capacity as proposed for *Arabidopsis AtRabG* overexpression lines [51]. Furthermore, Rab-GTP proteins are also involved in the delivery of new membrane materials such as ion channels or transporters that in turn can regulate Na^+^ and Cl^−^ transport across different membranes, as seen in mammalian epithelial cells where disruption is linked to cystic fibrosis [65].

While three of the chickpea accessions showed high Na^+^ accumulation in leaves, the other three (Genesis 836, Rupali and Yubileiny) appear to be ‘sodium excluders’ with substantially less Na^+^ in their leaves (Fig. 2). Exclusion of Na^+^ from leaves was about 10-fold more effective in Genesis 836 compared to Hattrick, ICC12726 and Slasher. In accordance with our observations, another study has shown that salt-tolerant Genesis 836 is an effective Na^+^ excluder, while the more salt-sensitive Rupali accumulated slightly higher levels of Na^+^. There were also different intercellular patterns of accumulation, whereby Genesis 836 compartmentalised Na^+^ into epidermal cells, enabling photosynthetic mesophyll cells to continue functioning, while Rupali accumulated Na^+^ in all cell types, to the detriment of photosynthesis rates and plant health [57, 58]. Redirection of Na^+^ to particular cell types in Genesis 836 may involve Rab-GTP proteins, which could traffic Na^+^ ions from the cytosol to the plasma membrane for expulsion in a cell-type specific manner, or increase Na^+^ intake into epithelial cells while inhibiting Na^+^ intake into mesophyll cells. In our experiments, Genesis 836 showed early, transient enhancement of *CaRabA2* expression while Rupali did not show any up-regulation of this gene at any time-point. Since all other *CaRab* genes showed similar responses between these two cultivars, *CaRabA2* genes may be important in regulating Na^+^ relocation between different cell types in Genesis 836 leaves. Further research is needed to determine which of the four genes from the *CaRabA2* clade is responsible for this and to explore the intercellular compartmentalisation in other chickpea accessions with highly responsive *CaRabA2* gene expression. It will also be important to determine which type of vesicle (i.e. SV or CCV) is regulated by *CaRabA2*. Whatever the answers to these questions, our study shows that the expression of *CaRabA2* is strongly correlated with Na^+^ accumulation in leaves.

**Fig. 6:**
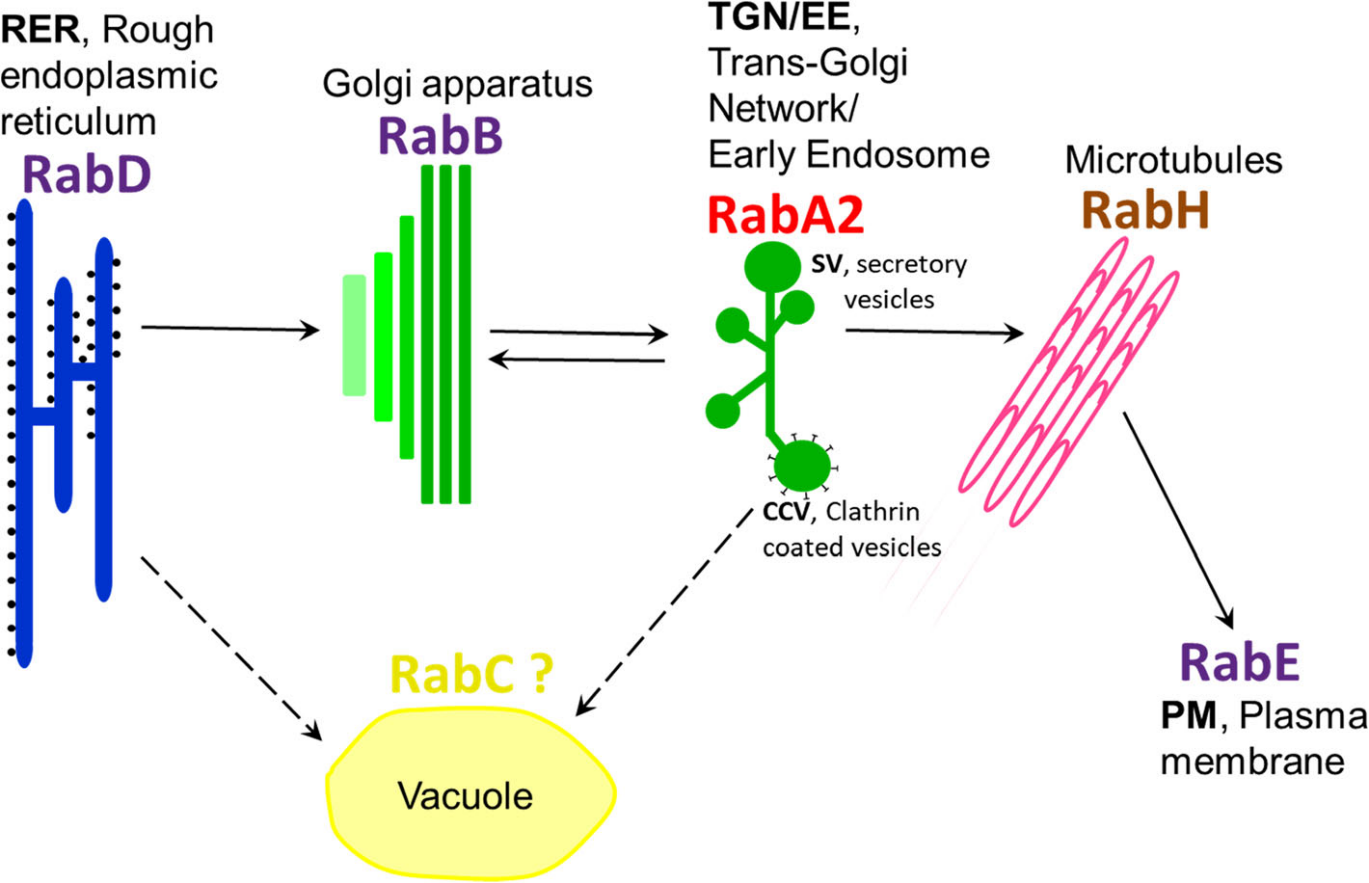
Model of CaRab protein locations and functions in plant cells. The model has been modified and adapted from those published earlier [10, 59]

Based on the new relationships uncovered in chickpea, we propose a slightly adjusted trafficking model for plant Rab-GTPs with a focus on salt stress (Fig. 6). In this model, we propose that membrane vesicles from the TGN/EE mobilise not only to the plasma membrane for exocytosis as directed by RabH and RabE, but also to the vacuole and/or plasma membrane, directed by RabA2. This could facilitate Na^+^ sequestration in the vacuole either through direct deposition of excess Na^+^ from the cytosol, or delivery of ion transport channels to the vacuole for enhanced Na^+^ uptake. Both of these processes are mediated via CCVs. Secretion at the plasma membrane for subsequent inter-cellular compartmentalisation via SVs, may also be important.

Despite the above observations, it should be noted that salinity tolerance per se (estimated as biomass production relative to control plants) was not correlated with either Na^+^ accumulation or the expression pattern of any of the six *CaRab* gene clades. This suggests that Na^+^ accumulation by itself cannot guarantee salinity tolerance in chickpea, but rather is only one of a number of different mechanisms, and many more genes are likely to be involved. Nonetheless, the Na^+^ accumulation in leaves and its strong correlation with *CaRabA2* gene expression, as well as the stable genotype-dependent expressions of *CaRabB*, *−D* and −*E* reported here, point to a role for intracellular trafficking *CaRab-*GTP genes in the salinity tolerant phenotype.

## Conclusions

*CaRab*-GTP gene expression patterns suggest that coordinated networks for intracellular trafficking operate under salt stress in chickpea, Generally, the different gene clades showed variable responses to stress in different chickpea accessions, but *CaRabB*, *−D* and *−E* had very similar expression profiles. This ‘trio’ may form the major trafficking route through the endosomal system. Since the expression of *CaRabA2* in response to salt was strongly correlated with Na^+^ accumulation in leaves, it may be more important in trafficking flexibility in response to environmental conditions. This confirms previous reports that indicate an important role of the *CaRab* gene family in the response of chickpea plants to salinity.

## Methods

### Plant material

Chickpea accessions, Hattrick and Slasher, were developed by Pulse Breeding Australia, licensed to Seednet and used as commercial cultivars in Australia. Seeds were generously provided by Helen Bramley, University of Sydney (Australia). Accessions Genesis 836 and Rupali were selected based on their contrasting salinity tolerance in previous studies [57, 66]. Genesis 836 originated from ICARDA (International Centre for Agricultural Research in the Dry Areas, Syria). Rupali is from the Department of Agriculture, WA and GRDC (Australia), licensed to AWB Seeds (Australia). ICC12726 is from the ICRISAT collection (India). Seeds of Genesis 836, Rupali and ICC12726 were kindly provided by Tim Sutton, SARDI-PIRSA, Adelaide (Australia). Yubileiny originated from Krasnokutskaya Breeding Station (Russia), and it is used as a standard for local field trials with chickpea accessions. Seeds were provided by Kazakh AgroTechnical University, Nur-Sultan (Kazakhstan).

### Plant growth and salt stress application

The experiment was carried out in 18 cm diameter pots lined with a plastic bag and filled with 2.6 kg of BioGro soil-mix, Adelaide (Australia). Seeds were germinated in Petri dishes with moisturised Whatmann paper for 5 days and seedlings were transplanted in pots (four seedlings per pot) with artificial inoculation of rhizobium (NodulAid, BASF, Australia). Plants were grown in pots with soil for 1 month in a controlled-temperature greenhouse with 25 °C/20 °C day/night temperature and 16 h LED Grow Lights (~PAR 500) (Heliospectra AB, Sweden). Pots were watered twice weekly on a portable scale, keeping soil moisture level consistent at 80% field capacity.

For salt stress, 150 ml of 190 mM NaCl was applied to each pot, with four increments, twice daily and over 2 days. Based on available soil moisture at 80% field capacity, the calculated level of salinity in the experiment reached 90 mM NaCl after last increment of the application and was maintained until the end of the experiment. In control pots, the same volume of tap-water without NaCl was used under the same schedule. No supplementary CaCl_2_ was added due to sufficient available calcium in the soil, and no symptoms of Ca deficiency were apparent in the control plants.

### Visual symptoms of salt stress and plant biomass production

Images were taken throughout the experiment to record visual differences in control and salt-treated plants, including chlorosis, browning and senescence of leaves, loss of shoot rigidity and decreased vegetation.

Fresh weight (FW) and dry weight (DW) were measured 1 month since last salt application, using whole shoots of eight plants in each of treatment group. Plants were cut at the base of the shoot, weighed, then dried in an 80 °C oven for 2 days before measuring dry weights. Samples were also taken for flame photometry from these plants.

### Flame-photometry for Na^+^ and K^+^ measurement in leaves

The youngest fully-developed leaves were collected from plants before harvesting for biomass after 1 month of salt exposure. Two leaves from the main shoot were pooled from each plant, making eight biological replicates. FW and DW were recorded, the latter after drying the samples for 2 days at 80 °C. Both DW and tissue sap (FW-DW) were used for calculation of Na^+^ and K^+^ levels in leaf samples following a previously published method for cereals [67]. Leaf samples were digested in 10 ml of 1% HNO_3_ at 80 °C for 4 hours. Concentrations of sodium and potassium ions were measured by Flame-photometer (Sherwood, UK, model 420) and expressed either as concentration (mM in plant sap) or as content (per g of DW) [68].

### RNA extraction, cDNA synthesis, semi-quantitative PCR and qPCR analysis

On days 5, 9 and 15 after the final NaCl application, four plants from each accession (four biological replicates) were randomly selected from each set of control and salt-treated pots. The two youngest fully developed leaves were snap-frozen in liquid nitrogen and stored at − 80 °C.

Frozen leaf samples were ground by nitrogen-cooled stainless steel ball bearings with vigorous vortexing. TRIzol-like reagent was used for RNA extraction following a previously described protocol [69]. Following DNase treatment (NEB Biolab, England), 2 μg of RNA was reverse transcribed with the use of Protoscript-II Reverse Transcriptase kit (NEB Biolab, England). Samples of cDNA diluted with water (1:10) were used for both semi-quantitative RT-PCR and qRT-PCR analyses. For semi-quantitative RT-PCR, cDNA from all samples were pooled together and used as a template in reactions containing 1.8 mM MgCl_2_, 0.2 mM dNTP, 0.25 μM of each primer and 1.0 unit of Go-Taq DNA polymerase (Promega, USA). Amplification was carried out with the following program: 94 °C for 2 min; 30 cycles of 94 °C for 10 s, 55 °C for 10 s, and 72 °C for 15 s; and final extension at 72 °C for 1 min. PCR products were visualised in a 1.5% agarose gel containing GelRed (Biotium, USA) alongside a 100 bp DNA Ladder (Bioline, USA), using a GelDoc system (BioRad, USA). Amplicon sizes varied between 88 and 210 bp and the information about primers is present in Additional file 1 (Table S1).

For qRT-PCR expression analysis, KAPA SYBR Fast Universal Mix (KAPA Biosystems, USA), was used in a Real-Time qPCR system CFX96 (BioRad, USA) according to a previously described protocol [70]. Expression levels of target genes were normalised relative to the geometric mean of two reference gene transcript levels: *Hsp90*, Heat shock protein 90 (GR406804) and *CaEf1α*, Elongation factor 1-alpha (AJ004960) [71]. Relative transcript levels of each *CaRab* gene used in the quantitative RT-PCR analysis are shown in Additional file 1 (Table S2, Fig. S4) for each of the six chickpea accessions, grown under control conditions.

### Statistical analysis

Excel 365 (Microsoft) and SPSS 25.0.0.0 (IBM) software packages were used to calculate and analyse means, standard errors and significance levels using unpaired *t*-test, ANOVA and Pearson’s correlation functions.

## Supplementary information

**Additional file 1:** **Table S1.** Sequences and information about primers used in the study. **Table S2.** Full list of chickpea Rab clades and their constituent genes, targeted by qRT-PCR. Including protein and gene IDs and calculated primer efficiencies for each primer set, based on nomenclature presented in [36]. **Figure S1.** Images of growing chickpea plants in non-stressed Controls and after 9 days since first time of salt application (90 mM NaCl) based on 80% of field capacity moisture. **Figure S2.** Images of growing chickpea plants in non-stressed Controls and after 1 month since first time of salt application (90 mM NaCl) based on 80% of field capacity moisture. **Figure S3.** Alignment results, primers design for *CaRab* genes and sequences of the *CaRab* gene accessions. **Figure S4.** Relative transcript levels of each *CaRab* gene used in the quantitative RT-PCR analysis, in each of the six chickpea accessions, grown under control conditions.

## Abbreviations

CCV: Clathrin-coated vesicles
CORVET: Class C core vacuole/endosome tethering
DW: Dry weight
EE: Early endosome
FW: Fresh weight
GDI: Guanidine diphosphatase dissociation inhibitor
GDP: Guanidine diphosphatase
GEF: GDP/GTP exchange factors
GEP: GDP/GTP exchange proteins
GTP: Guanidine triphosphatase
HOPS: Homotypic fusion and protein sorting
REP: Rab escort protein
RER: Rough endoplasmic reticulum
ROS: Reactive oxygen species
SV: Secretory vesicles
TGN: *Trans*-golgi network
TRAPPII: Transport protein particle II
