# Supplementary material for: Salt-induced expression of intracellular vesicle trafficking genes, CaRab-GTP, and their association with Na+ accumulation in leaves of chickpea (Cicer arietinum L.)
Source: BMC Plant Biol. 2020 Oct 14;20(Suppl 1):183. doi: 10.1186/s12870-020-02331-5 (PMC7557026; doi:10.1186/s12870-020-02331-5)

# Additional file 1

**for the paper Sweetman et al. ‘Salt-induced expression of intracellular vesicle trafficking genes, *CaRab*-GTP, and their association with $Na^+$ accumulation in leaves of chickpea (*Cicer arietinum* L.)’**

**Table S1.** Sequences and information about primers used in the study.

Degenerate nucleotides are indicated in yellow.

| Name | Sequence 5’-3’ | Amplicon size (bp) |
|---|---|---|
| CaRabA1-Fq | RAARGARYTDMGRRATCACACA | 193 |
| CaRabA1-Rq | MGTVVRMACYTCRGYRAANGCA | |
| CaRabA2-Fq | TTCBAACATTGTKATYATGATG | 210 |
| CaRabA2-Rq | TGDGCWGCAAGTGCTTTTTTAC | |
| CaRabA3-Fq | AGTGTAYGACATAACWAAAAGA | 150 |
| CaRabA3-Rq | YTATTAATCTCTTCAARCAATT | |
| CaRabA4-Fq | AGTTTAYGACATNACAAARCGT | 145 |
| CaRabA4-Rq | YDABBHKRTATATTTCKGTHARA | |
| CaRabA5-Fq | ATYTKRAKRATRYVAGRGMRGTG | 161 |
| CaRabA5-Rq | DRACYTTBCKGCTKAHDWTRTT | |
| CaRabA6-Fq | RGCARTGYTRGTGTATGACATA | 159 |
| CaRabA6-Rq | CCYTCTTCTTTMTCAACTTSTC | |
| CaRabB-Fq | TCAYTTRGCWAGYTGGTTGGAAGA | 194 |
| CaRabB-Rq | AWGCCTCTTCWACRTTYTGAGC | |
| CaRabC-Fq | GTTAAARCTTRCYATTTGGGA | 88 |
| CaRabC-Rq | AATTATTCCTTGTGCWCCTC | |
| CaRabD-Fq | TGAAATTGACCGHTATGCMAGT | 146 |
| CaRabD-Rq | TWGCACTTGTYTCCATRAAAGG | |
| CaRabE-Fq | GTGGWGCYATGGGHATHTTGC | 165 |
| CaRabE-Rq | TWGGHACAGCCCTTTTVCTTTC | |
| CaRabF-Fq | RAYKMWRCAACWGTSAARTTTG | 199 |
| CaRabF-Rq | CTAATGCCATCACTATATCAGG | |
| CaRabG-Fq | TTRACMAYTGGMRNGARGART | 190 |
| CaRabG-Rq | YCYTCTTTTGCAGAKGTCTCAA | |
| CaRabH-Fq | ACAARCTSGTTTTCTTAGGYGATC | 167 |
| CaRabH-Rq | CDGTATCCCAHARCTGCAGHCG | |
| CaHsp90-F | GCAGCATGGCTGGTTACATGT | 63 |
| CaHsp90-R | TGATGGGATTCTCAGGGTTGA | |
| CaEf1α-F | TCCACCACTTGGTCGTTTTG | 64 |
| CaEf1α-R | CTTAATGACACCGACAGCAACAG | |

Standard nucleotide coding system:
R = A or G; Y = C or T; M = A or C; K = G or T; S = C or G; W = A or T; H = A, C or T; B = C, G or T; V = A, C or G; D = A, G or T; N = A, C, G or T.
N/A indicates primers that were not used for qPCR due to insufficient amplification or poor primer efficiency.

**Table S2.** Full list of chickpea Rab clades and their constituent genes, targeted by qRT-PCR. Including protein and gene IDs and calculated primer efficiencies for each primer set, based on nomenclature presented in [36]. Asterisks are part of the gene name classification system, indicating very similar polypeptide sequences between isoforms of the same name.

| Gene clade primer set | Genes targeted by primer set | Protein accession (NCBI) | KEGG IG (Chickpea portal) | Primer efficiency (%) |
|---|---|---|---|---|
| CaRabA2-Fq/Rq | *CaRabA-2a* | XP_004485429 | 101495904 | 84 |
| | *CaRabA-2b* | XP_004503210 | 101503536 | |
| | *CaRabA-2b** | XP_004490850 | 101512788 | |
| | *CaRabA-2d* | XP_004507156 | 101512110 | |
| CaRabB-Fq/Rq | *CaRabB-1b* | XP_004489550 | 101515168 | 96 |
| | *CaRabB-1c* | XP_004486381 | 101501307 | |
| | *CaRabB-1c** | XP_004510833 | 101496042 | |
| CaRabC-Fq/Rq | *CaRabC-1a* | XP_004498372 | 101496214 | 92 |
| | *CaRabC-1b* | XP_004502943 | 101488438 | |
| | *CaRabC-1c* | XP_004503936 | 101490080 | |
| | *CaRabC-2a* | XP_004515929 | 101497183 | |
| | *CaRabC-2a** | XP_004496130 | 101498490 | |
| CaRabD-Fq/Rq | *CaRabD-1* | XP_004485428 | 101495577 | 87 |
| | *CaRabD-2a* | XP_004492924 | 101506934 | |
| | *CaRabD-2a** | XP_004515343 | 101514122 | |
| | *CaRabD-2c* | NP_001265926 | 101496365 | |
| CaRabE-Fq/Rq | *CaRabE-1a* | XP_004495000 | 101497052 | 87 |
| | *CaRabE-1a** | XP_004487032 | 101515594 | |
| | *CaRabE-1b* | XP_004494002 | 101504780 | |
| | *CaRAbE-1c* | XP_004497298 | 101491866 | |
| | *CaRabE-1c** | XP_004505885 | 101506447 | |
| CaRabH-Fq/Rq | *CaRabH-1d* | XP_004508989 | 101507228 | 89 |
| | *CaRabH-1d** | XP_004511760 | 101496604 | |
| | *CaRabH-1e* | XP_004502420 | 101492440 | |
| | *CaRabH-3e** | XP_004504075 | 101507522 | |

**Figure S1.** Images of growing chickpea plants in non-stressed Controls and after 9 days since first time of salt application (90 mM NaCl) based on 80% of field capacity moisture. **(A)** Genesis 836; **(B)** Hattrick; **(C)** ICC12726; **(D)** Rupali; **(E)** Slasher; **(F)** Yubileiny.

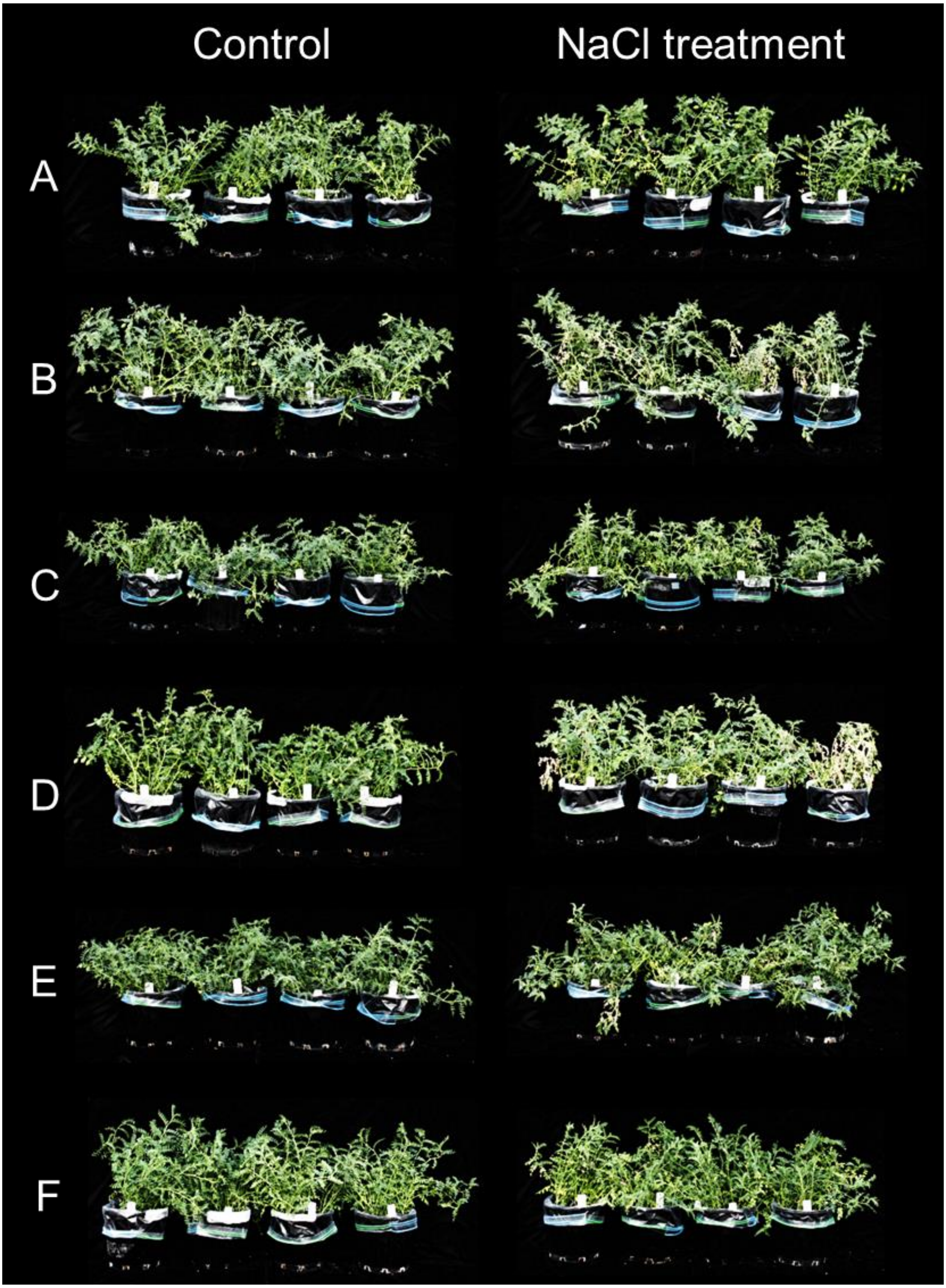

**Figure S2.** Images of growing chickpea plants in non-stressed Controls and after one month since first time of salt application (90 mM NaCl) based on 80% of field capacity moisture. **(A)** Genesis 836; **(B)** Hattrick; **(C)** ICC12726; **(D)** Rupali; **(E)** Slasher; **(F)** Yubileiny.

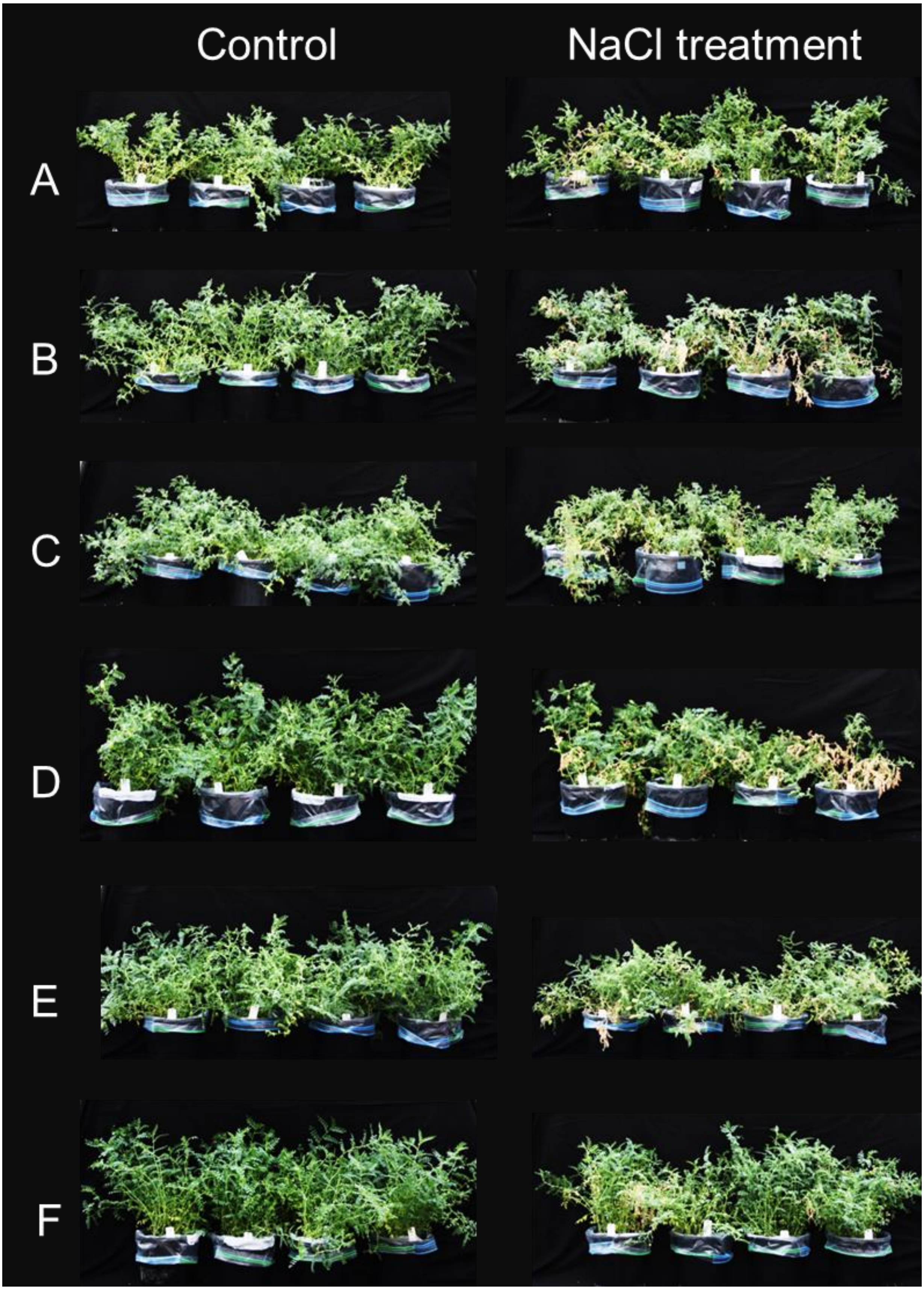

**Figure S3.** Alignment results, primers design for *CaRab* genes and sequences of the *CaRab* gene accessions. Primers indicated in different colour targeted different *CaRab* clades, and degenerate nucleotides are indicated in contrast colour. The information about primers is summarised in Table S1.

```
RabB-1c*      aaagaaaatcctacgcatatatttcaaatacatattaatcggtgacactggagttggaa    106
RabB-1b       -----atgtcgtacgattacctcttcaagtacatcatcatcggcgacacaggtgtaggga   55
RabB-1c       -----atgtcttacgcgtacctcttcaaatacatcatcatcggtgacactggagttggaa   55
RabA-5a       aagagaagaccgaggattacctttcaaaattgttttaattggtgattcagctgttggga    79
RabA-5b       gtgaaggaggtgaagagtacttgttcaagatcgtgttaatcggcgactcagcagtaggaa   73
RabA-5e       aaggaggaggagaagagtatctcttcaaagtcgttataatcggtgactccgcagttggta   79
RabA-3        tagaagagaaaatagattatgtgtttaaggttgtggtgataggagactcagcagtaggaa   97
RabA-3*       tggaggagaaaatagattatgtgtttaaggtgattgtaattggggattcagcagtaggaa   109
RabA-4c       ctgatgacgatattgattacatgtttaaggttgtgttaattggagactctggagttggaa   82
RabA-4a       caaaccagaaaatagactacgttttcaaggtagttctaatcggtgattctgcggtaggga   88
RabA-4a*      caaaccagaggatagattatgtattcaaggtggttttgataggggattcagcagtgggga   91
RabA-4d       acaacttgaagatcgattacgtattcaaggttgtattgattggtgattcagcagttggaa   109
RabA-4d*      acaatcaaaagatcgattacgttttcaaagttgtattaattggggactctgctgtcggca   82
RabA-6a       ttgatgaggagtgtgattacctcttcaaagctgttttaattggagattcaggagttggga   73
RabA-6a*      ttgatgaagagtgtgattacctgttcaaggcagtattgatcggagactctggagttggaa   73
RabA-1g       ccgacgaagaatatgattacttgttcaagttggttttgatcggtgactccggtgttggaa   70
RabA-1g*      ccgacgacgactacgactacctatttaaggtggttctgatcggagactccggcgtcggta   76
RabA-1f       ccgatgacgattacgattatctattcaaagtggttttgatcggagactcaggtgttggta   76
RabA-1f*      ctgatgatgattatgattatctgttcaaggttgttctgattggtgattctggtgttggaa   76
RabA-1c       cagatgatgaatacgattacctttcaagctggttttgattggtgattctggagttggca    76
RabA-1d       cagatgatgagtacgattacttgttcaaacttgttctgattggcgattccggtgttggta   76
RabA-1b       cagacgatgactacgactatctattcaaggtagttctgatcggcgactccggtgtcggca   76
RabA-1b*      cggatgacgactacgactacctttcaaagtagttctaatcggtgattccggcgtaggga    76
RabA-2a       gagaagaagaatacgattatctgttcaaggtagtactgatcggcgattctggcgttggta   73
RabA-2d       tggatcatgagtatgattacttgttcaagatcgttttgatcggagattctggtgttggaa   73
RabA-2b       tagaccatgaatatgactatctcttcaagatcgtactcattggtgattctggtgttggaa   73
RabA-2b*      tagatcatgaatacgattatctattcaaggttgttttaattggggactctggtgttggaa   73
RabE-1a*      ccggtgctgatcccgattatctcataaagcttcttttgatcggagatagtggtgttggaa   88
RabE-1a       ctcgttccgattacgattatctcattaagcttctccttatcggtgatagcggtgtgggta   73
RabE-1c       ctcgtgccgattacgattacctcatcaagcttcttcttatcggcgacagtggtgtgggga   82
RabE-1c*      ctcgtgccgattacgattatctcattaagcttctcttgatcggcgatagcggtgttggaa   82
RabD-1        tgagcaacgaatacgattacctatttaaggttcttataatcggagactcctccgttggaa   61
RabD-2c       tgaatcccgaatatgactatttgttcaagcttttgttgattggagattctggtgtgggca   61
RabD-2a       tgaatcccgagtatgattatctgttcaagcttcttcttattggagactctggtgttggca   61
RabD-2a*      tgaatcccgagtatgattatctgttcaagctccttcttattggagactctggtgttggta   61
RabC-2a       gcagcagtagctatgatctctctttcaagatcttgttgattggtgattctgctGttggaa   187
RabC-1a       gtcacgccgaattcgattacttattcaagcttctgttaattggtgattctggagtcggca   296
RabC-1c       ggcagcaagaattcgactatttgttcaagttgttaatgattggagactctggtgttggaa   76
RabC-1b       cgagtcaggaattcgattatttgttcaagttgttgatgattggggactctggagttggca   73

RabH-1d       cgccagtttcagctcttgctaagtacaaattggttttcttaggcgatcaatccgtcggta   64
RabH-1d*      cgccagtgtcagctcttgcaaagtacaagctggtttttttaggtgatcaatctgtgggta   64
RabH-1e       caaccgtttcacctctcgccaaatacaaactcgttttcttaggtgatcaatcggttggca   64
RabH-1e*      cgacggtttcccctctcgccaaatacaagctcgttttcttaggcgatcaatcggttggta   64
                                Forward ACAARCTSGTTTTCTTAGGYGATC

RabG-3f       tgccgtcccgaagaagaactctattgaaggtcatcattctcggtgacagcggggtgggga   61
RabG-3d       tggcttctcgccgccgcatgttattgaaagtcattatcctcggagatagcggggttggca   61
RabG-3c       ------------------------------------------------------------   0
RabG-3e       tggcttctcgccgccgcatgttgttaaaagttataatcctcggtgatagtggggttggta   61
RabG-3b*      tttcacatagaaagagaactttgcttaaggttatcgtccttggagatagtggggtgggga   67
RabG-3a       tgtcattgcgcagacgaaccttgctcaaggtcattgtcctcggcgatagcggggttggaa   61
RabG-3b       tgtcattacgcaaacgaaccttgctgaaagtgattgttctaggagacagcggggttggaa   61
RabF-1        gtcaagacgctaagaatcttcgcgtcaagcttgtcctcttaggcgattctggtgttggta   133
RabF-2b       tggcaaggaataagagtttacaagccaaactggtacttctgggggacatgggaactggga   61
```

```
RabB-1c*      aatcatgtctccaacttcaattcaccgaccaccacttccaacctttccacgacgtaacaa    166
RabB-1b       aatcttgtctgctccttcagttcaccgacaagaggtttcaacctgttcatgatcttacaa    115
RabB-1c       agtcgtgtcttctactacagttcactgacaagcgctttcaacccgtccatgacttgacca    115
RabA-5a       aatcaaatttacttgcaagatttgccagggatgaattttatcctaattcaaagtcaacta    139
RabA-5b       aatcgaacctactttcacgattcgcacgaaacgaattcgactcgaattcaaaagcaacaa    133
RabA-5e       aatcaaatctactctctagatacgctcgtaacgagttcaatcttcactccaaagctacaa    139
RabA-3        agactcaaatactgtcaaggtttgcaaagaatgagttctgctttgactcaaaatcaacca    157
RabA-3*       agacacaaatactgtcaaggttttcaaagaatgagttctgttttgactctaagtcaacta    169
RabA-4c       aatctcagcttttaaatcggtttgttagaaatgaatttcacatgaaatctaaagctacaa    142
RabA-4a       agtctcaaatacttgctcgatttgcgagaaacgacttcagcttagactctaaagccacaa    148
RabA-4a*      aatcacagatattagctagattttctaggaacgagttcagtttagattctaagtccacga    151
RabA-4d       aaactcaactactcgctcgttttgcaaggaacgaatttaacgttgattcgaaggccacaa    169
RabA-4d*      aaacgcaacttctcgcacgattttcaaggaatcaattcaacgtcgattctaaagccacca    142
RabA-6a       aaacaaatcttatttcaaggtttgcaaaagatgaatttcgacttgattccaaaccaacca    133
RabA-6a*      aatcaaatctgctttcaagatttgcaaaagatgaattcaggttggattcaaaaccaacta    133
RabA-1g       agtccaaccttttgtctagattcacccgaaatgaattcaacttggaatccaaatccacca    130
RabA-1g*      aatccaacctcttgtcacgattcaccaaaaatgaattcagtttggaatctaaatccacca    136
RabA-1f       aatcaaatcttctttcgaggttcacaaagaatgaattcagcctcgaatctaaatccacca    136
RabA-1f*      aatccaaccttttgtctagattcaccaagaatgaatttagccttgaatcaaaatccacca    136
RabA-1c       aatctaaccttctttccaggttcaccaaaaacgagttcaatttggagtccaagtccacca    136
RabA-1d       aatccaatttgctttctaggttcactaggaatgagttcaatttggaatctaaatccacca    136
RabA-1b       aatcgaatcttctctcgcgtttcacgaagaacgagttcaaccttgagtccaaatccacca    136
RabA-1b*      agtcaaaccttctttcaaggttcaccaagaacgagttcaacctcgaatctaagtccacca    136
RabA-2a       aatccaatcttctctcccgattcactcgcaatgagttttgtctcgagtccaagtccacta    133
RabA-2d       aatctaacatcctttccaggtttactcgaaacgagttttgtttggagtccaaatctacta    133
RabA-2b       aatcaaatatactctccaggtttacccgaaatgagttctgtttagaatctaaatctacca    133
RabA-2b*      aatccaatatcctttctcgttttacaagaaatgagttttgtttggaatctaaatctacca    133
RabE-1a*      agagttgccttcttctacgattctccgatggttctttcacaactaattttattacaacct    148
RabE-1a       aaagttgccttctattgcgtttctcagatggatcattcacaactagttttataacaacca    133
RabE-1c       agagttgtcttcttttgagattttccgatggttccttcacaaccagttttatcaccacca    142
RabE-1c*      aaagttgccttcttttgcgtttttctgatggttccttcaccaccagtttcatcacaacta    142
RabD-1        agtcttgcttgcttctcagattcgccgatgactcctatgtcgatacctacattagtacca    121
RabD-2c       agtcatgtctcctcctgaggtttgctgatgattcataccttgacagctatatcagtacaa    121
RabD-2a       aatcatgccttcttctgagatttgctgatgattcttacattgacagctacataagcacca    121
RabD-2a*      aatcatgccttcttctaagatttgctgatgattcatacattgatagctacataagcacca    121
RabC-2a       aaagcagcttaattcttagcttcatctctgcttctgttgaag---atctttcccccActa    244
RabC-1a       aaagcacgctgcttctcagattcacctctgataatttcgagg---atctttctcctacca    353
RabC-1c       aaagtagttttcttctcagattcacctccgatgatttcgaag---atctctcccccacca    133
RabC-1b       agagtagtctcctcctctgtttcacctctgatgctttcgaag---atctatccccaacaa    130
RabH-1d       aaaccagcatcatcactcgcttcatgtacgacaaattcgataacacctatcaggctacaa    124
RabH-1d*      aaaccagcatcattactcgcttcatgtatgataaatttgacaacacttatcaggctacaa    124
RabH-1e       agaccagcatcatcactcgtttcatgtatgacaaattcgatactacctatcaggccacta    124
RabH-1e*      aaaccagcatcatcacccgtttcatgtacgacaaattcgacacaacctatcaggctacta    124
RabG-3f       agacatctttgatgaatcaatatgtgaataagaagtttagcaatcagtataaggcaacca    121
RabG-3d       aaacatcactcatgaatcagtatgtgaatagaaagtttagtaatcagtataaggctacca    121
RabG-3c       ------------------------------------------------------------    0
RabG-3e       aaacatcattgatgaatcagtatgtgaatcgtaagtttagtaatcagtacaaggctacca    121
RabG-3b*      aaacgtctttgatgaatcaatatgtttataagaaatttagccaacagtataaagccacga    127
RabG-3a       aaacctcgttgatgaatcaatatgtgcacaacaagtttagtcaacaatataaggctacta    121
RabG-3b       agacttctttgatgaatcaatatgtgcacaacaagtttagtcagcaatataaagctacaa    121
RabF-1        agagctgtattgttctgagatttgttcgtggtcagtttgacccgacatcgaaggtaactg    193
RabF-2b       agacgagtttggttcttagatttgtcaaaggtcaattttcggattaccaggaatcgacaa    121
```

```
RabB-1c*      ttggtgttgaattcggcaccaggacaatcaac---attgaaaaaattccaataaagttac    223
RabB-1b       ttggtgttgagtttggtgctcgtatgctcacc---attgattctaggcctattaagcttc    172
RabB-1c       ttggtgttgaatttggtgcaaggatgatcact---attgataataagccaatcaagttgc    172
RabA-5a       taggagtagagttccaaactcagaaaatggaa---attaacggaaaggaagttaaagcgc    196
RabA-5b       tcggcgttgaatttcagacgcaaatggtggaa---atcgacggtaaagaagtgaaggcgc    190
RabA-5e       tcggtgttgagtttcaaactcagagcttagaa---atcgattccaaagaagttaaggctc    196
RabA-3        ttggagttgaatttcaaactaaaactgtcaca---ataaatggtaaactcatcaaagcac    214
RabA-3*       ttggagttgaatttcaaactaggactgttatt---attaatggtaaagttatcaaagctc    226
RabA-4c       ttggtgttgaatttctgactaagacggttctc---atggatcacaaacatgtcaaggctc    199
RabA-4a       tcggcgtcgagtttcagactcgcactttggtc---attcaacataagagcgttaaagctc    205
RabA-4a*      tcggagttgaatttcaaacacgtactttagtc---atcgatcataagaccgtcaaggctc    208
RabA-4d       ttggggtcgaatttcaaaccaaaactttaatt---attgataataaaactattaaggctc    226
RabA-4d*      tcggcgtcgagtttcagaccaaaactcttata---atcgataataaaaccgttaaggctc    199
RabA-6a       taggtgtggaatttggttacaagaacatcaaa---gttagagacaaactcatcaaagcac    190
RabA-6a*      ttggagttgaatttgcttacagaaacatcaaa---gttagagacaaactcatcaaagctc    190
RabA-1g       tcggcgttgaattcgctactcgcagtgttcgt---attcttgacaaacttgtcaaagccc    187
RabA-1g*      ttggtgttgaattcgcaacacgcagcatacac---gtcgatgataagattataaaggcac    193
RabA-1f       ttggcgttgagttcgctaccagaagcattcgt---gtagatgataaggttgtcaaggctc    193
RabA-1f*      ttggtgttgaatttgcaaccagaagcattagg---gttgatgataaggttgtcaaggctc    193
RabA-1c       taggtgtcgaattcgccaccaaaactttgaat---attcatgctaaaatcatcaaggctc    193
RabA-1d       taggtgttgagtttgctaccaagagtttgact---attgattccaaagttatcaaggctc    193
RabA-1b       ttggtgttgagttcgctactcgtactttgaat---gttgattctaaagtcatcaaggctc    193
RabA-1b*      ttggcgtcgagttcgccacacgcacccttaac---gtcgacaccaaagtcgttaaatctc    193
RabA-2a       tcggcgttgaattcgccacccgtaccctcaag---gtcgagtcaaaaaccgttaaagctc    190
RabA-2d       ttggagttgaattcgccaccagaacccttcag---gtagagggaaagactgtaaaggcac    190
RabA-2b       ttggtgttgaattcgctaccagaacaatacag---gtggaagggaaaacagtgaaagcac    190
RabA-2b*      ttggtgttgaatttgcaacaaggacattacag---gtagagggaaagacagtaaaggcac    190
RabE-1a*      taggcattgattataaaaatagagccattcag---ctggatggaaaaaagatgatgatcc    205
RabE-1a       tcggcattgatttcaaaataaggacaatagag---cttgatggaaagcgaatcaaattgc    190
RabE-1c       ttggaattgatttcaagataagaaccattgaa---cttgatggcaaacgcattaagttac    199
RabE-1c*      taggcattgattttaagataagaactattgag---cttgatggcaaacggatcaagctcc    199
RabD-1        ttggcgtcgatttcaaaatcagaactgtggaa---ttggaagggaaaaccgtcaagctgc    178
RabD-2c       ttggagtggactttaaaattcgcactgttgag---caagacgggaagaccattaaacttc    178
RabD-2a       ttggagttgattttaaaatacgcaccgttgag---caggatgggaagaccattaagctcc    178
RabD-2a*      tcggagttgattttaaaatacgaactgttgag---caggatgggaagacaattaaactac    178
RabC-2a       ttggtgttgattttaagatcaagcttctaaca---gtaggtggcaagagattgaaactaA    301
RabC-1a       tcggtgtagacttcaaagtgaaatatgttaca---attggtggaaaaaagttaaaacttg    410
RabC-1c       ttggtgttgattttaaggtcaaatatgtcatg---atgggggtaaaaaactcaagcttg    190
RabC-1b       ttggtgttgattttaaggtcaaatatgttact---atggatggtaaaaagttgaagcttg    187

RabH-1d       tcggcattgattttctatcaaaaaccatgtat---cttgaagatcgaactgttcgactgc    181
RabH-1d*      ttggtattgatttctgtcgaaaactatgtat---cttgaagatcgaactgttcggctgc    181
RabH-1e       ttggtattgatttttgtcaaaaacaatgtac---cttgaagatcgaacagttcgattgc    181
RabH-1e*      ttggtatcgatttttgtcaaaaacaatgtac---cttgaagatagaactgttcgtctgc    181
                                                                 CGDCTGC

RabG-3f       ttggagcggatttcttaaccaaagaagtgcaa---tttgaagataggcttttcactttac    178
RabG-3d       ttggcgctgatttccttaccaaggaagttcaa---tttgaagataggttgttcacattgc    178
RabG-3c       ------------------------------------------------------------    0
RabG-3e       ttggtgccgatttcctcaccaaggaagttcaa---tttgaagacaggttgttcacattgc    178
RabG-3b*      ttggagctgatttgttacaaaggagatacta---gttgacgacaaactagtaaccttgc    184
RabG-3a       ttggtgctgatttgtcactaaagaactccaa---attgacgacagactcgtcactctac    178
RabG-3b       ttggtgctgatttgtcactaaagaactgcag---atcgatgacagactcgttactctac    178

RabF-1        ttggagcttctttcttgtcacaaacaatcgctcttcaagactctacaacagtcaagtttg    253
RabF-2b       ttggagcagcatttttactcaggttttgtca---ttaaatgaagcaactgtgaaatttg    178
                                            Forward RAYKMWRCAACWGTSAARTTTG
```

```
RabB-1c*     aattatgggacacagcgggtcaag---------------aaaattttagatcaattacaa    268
RabB-1b      agatatgggacactgctggacaag---------------agtcttttagatccatcacta    217
RabB-1c      aaatatgggatacggcgggtcaag---------------aatccttcagatctattacaa    217
RabA-5a      agatatgggacacagccgggcaag---------------agaggttcagagctgttacgt    241
RabA-5b      agatctgggatactgctggtcaag---------------aacgtttcagagctgttactt    235
RabA-5e      agatttgggatactgccggtcaag---------------aacgcttccgtgctgtaacct    241
RabA-3       agatctgggatactgctggccaag---------------aaaggtacagagcagtgacaa    259
RabA-3*      agatctgggatactgctggccaag---------------aaaggtacagggcagtaacaa    271
RabA-4c      agatttgggatactgctggtcaag---------------aaaggtaccaagcaattacaa    244
RabA-4a      agatctgggacactgctggccaag---------------aacgttatagagcagttacaa    250
RabA-4a*     agatctgggacactgctggtcaag---------------aacgatacagagcagttacaa    253
RabA-4d      aaatatgggatactgcaggtcaag---------------aaagatacagggcagttacta    271
RabA-4d*     aaatatgggacacggctggtcaag---------------aaaggtacagagcagtaacta    244
RabA-6a      aaatatgggacactgctggccaag---------------agaggtttagagctatcacaa    235
RabA-6a*     aaatatgggacactgctggtcaag---------------aaaggttcagagcaatcacaa    235
RabA-1g      agatttgggacactgctggtcaag---------------aaagatatcgtgcaatcacaa    232
RabA-1g*     aaatttgggataccgcaggacaag---------------aaagatatcgagcgatcacaa    238
RabA-1f      agatttgggatactgccggtcaag---------------aaaggtaccgagcaattacaa    238
RabA-1f*     agatttgggatactgctggccagg---------------aaaggtaccgagcaattacaa    238
RabA-1c      agatttgggacactgctggacaag---------------aaaggtaccgtgccattacca    238
RabA-1d      agatttgggatactgctggtcagg---------------aaaggtaccgtgccattacta    238
RabA-1b      agatctgggatactgctggtcaag---------------agaggtatcgtgccatcacca    238
RabA-1b*     agatctgggacaccgccggtcagg---------------aaaggtatcgagcgatcacca    238
RabA-2a      agatatgggacaccgccggacagg---------------aacgttacagagcgatcacca    235
RabA-2d      agatatgggacacagcaggtcagg---------------aacggtaccgtgctattacca    235
RabA-2b      aaatatgggacactgcaggtcaag---------------agaggtacagagcaatcacaa    235
RabA-2b*     aaatatgggacactgctggtcaag---------------aaaggtacagagcaattacaa    235
RabE-1a*     aagtttgggatactgcaggtcagg---------------agcggttccgaacaattacaa    250
RabE-1a      aaatatgggatacagctggtcaag---------------agcgtgttcgaactattacaa    235
RabE-1c      aaatctgggatacagctggtcagg---------------agagattccgaactattacta    244
RabE-1c*     aaatttgggatactgcagggcagg---------------agcggtttcgaacaattacaa    244
RabD-1       agatttgggatacggctggacagg---------------agcgattcaggactataacta    223
RabD-2c      aaatttgggacactgctggtcaag---------------aacgtttccggactatcacta    223
RabD-2a      agatttgggatactgctgggcaag---------------aacgatttaggacaatcacca    223
RabD-2a*     agatttgggatactgccgggcaag---------------aacgatttaggacaataacca    223
RabC-2a      ctatttgggacactgctgggcagg---------------aaaggttcagaacactaacta    346
RabC-1a      ctatttgggacacagctggacaag---------------aaaggtttagaacacttacca    455
RabC-1c      ccatttgggataccgctggtcagg---------------agagatttagaacactcacaa    235
RabC-1b      ccatttgggatacagctggtcagg---------------agagatttagaacactaacaa    232

RabH-1d      agttatgggatacagctggacagg---------------agagatttagaagtctaattc    226
RabH-1d*     agttgtgggatacagctgggcagg---------------aaagatttagaagtcttattc    226
RabH-1e      agctttgggataccgcaggacaag---------------aaagatttagaagtctgattc    226
RabH-1e*     agctttgggatactgcaggccaag---------------aaagatttagaagtcttattc    226
             AGYTDTGGGATACH Reverse

RabG-3f      agatttgggatacagctggccagg---------------aaaggttccaaagcctaggag    223
RabG-3d      agatctgggatactgctggtcagg---------------agaggtttcaaagtcttggcg    223
RabG-3c      -----------atgcatactgaaaaaatagtgtgcttttgggagttattaagtcttggcg    49
RabG-3e      agatttgggatactgctggnnnnnnnnnnnnnnnnnnnnagcggtttcaaagtcttggtg    238
RabG-3b*     aaatttgggatacagcaggacagg---------------aaaggtttcatagtcttggag    229
RabG-3a      aaatatgggacactgctggacaag---------------agagattccaaagtcttggag    223
RabG-3b      aaatatgggacactgcagggcaag---------------agagatttcaaagccttggtg    223
RabF-1       aaatatgggataccgctggtcaag---------------agaggtatgctgcattggcac    298
RabF-2b      atatatgggacacagcagggcagg---------------aacgataccacagtttggctc    223
```

```
RabB-1c*        gatcatattatagagatacaacatgtgcattactagtttatgatgtaacaaggagagaaa    328
RabB-1b         gatcttactacagaggagcagcaggggcacttctagtttatgacattacaaggagagaga    277
RabB-1c         ggtcgtattacagaggggctgcaggtgcactgcttgtttatgatataaccaggagggaga    277
RabA-5a         ctgcttattataggggtgcagttggagcacttctggtatatgacattagcaggcgccaaa    301
RabA-5b         ctgcttactatagaggtgctgttggtgctcttgttgtttatgatattagtaggagaggta    295
RabA-5e         ccgcttattacagaggcgctgtcggtgctcttgttgtttatgatattagccggagaacta    301

RabA-3          gtgcatactacagaggagcattaggggcaatgctagtgtacgacataactaaaagacaaa    319
RabA-3*         gtgcatactacagaggagcattaggggccatgttagtgtatgacataacaaaaagacaat    331
                                          Forward AGTGTAYGACATAACWAAAAGA

RabA-4c         ctgcatattacagaggtgcaactggtgcattactaacatatgacgtaaccaagcgccatt    304
RabA-4a         gtgcatactacaggggtgctgttggggcaatgttggtttatgacattacaaaacgtcaga    310
RabA-4a*        gtgcatactacaggggtgctgtaggggcaatgttagtttatgacatcacaaaacgtcaga    313
RabA-4d         gtgcttactatcgaggtgcagttggggcaatgttagtttacgacatgacaaagcgtcaat    331
RabA-4d*        gtgcgtattatcgcggagcagttggagcaatgttagtttacgacatgacaaagcgtcagt    304
                                          Forward AGTTTAYGACATNACAAARCGT

RabA-6a         gctcatactatagaggagccttaggggcaatgctagtgtatgacataactagaagaacaa    295
RabA-6a*        gctcgtactatagaggagccttgggagcagtgttggtgtatgacataacaaggcgatcaa    295
                                 Forward RGCARTGYTRGTGTATGACATA

RabA-1g         gtgcttactaccgtggagccgttggtgcactgcttgtgtacgatactacaaggcacgtga    292
RabA-1g*        gtgcatattatcgaggcgctgtcggtgcattgcttgtgtatgatgttacaaggcatgtaa    298
RabA-1f         gtgcttattatcgtggagctgttggcgctttattagtctatgatgttactcgtcgtgtta    298
RabA-1f*        gtgcatattatagaggagctgttggtgctttactagtgtatgatgttacacgccatgtta    298
RabA-1c         gtgcttactaccgaggagctgtcggggccttacttgtctacgatgtaacccgcagttcaa    298
RabA-1d         gtgcctactatcgaggagctgttggtgccttacttgtttatgatgtcacacggcgggcta    298
RabA-1b         gtgcttactatcgcggggcagtcggtgcacttcttgtatatgacgtcacacgccatgcaa    298
RabA-1b*        gtgcttattatcgtggggctgtaggtgcacttcttgtatacgatgtcacacgccatgcaa    298
RabA-2a         gcgcctactatcgcggtgctcttggtgctcttcttgtctacgatgtgaccaaaccaatta    295
RabA-2d         gtgcctattatagaggagctgtgggtgctctccttgtatatgacataactaagaggcaaa    295
RabA-2b         gtgcatattacagaggagctgttggtgccttattagtgtacgacataatcaagagacaaa    295
RabA-2b*        gtgcttactatagaggagctgttggtgcattattggtttatgacataacaaagagacaaa    295

RabE-1a*        atacttactatcgcggtgctatgggagtattgctagtctacgatgttactgacgaatttt    310
RabE-1a         ctgcttactaccgtggagccatgggcattttgcttgtgtatgatgtcactgatgagtcgt    295
RabE-1c         cagcttattaccgtggagccatgggtatcttgctggtttatgatgttactgatgaagcat    304
RabE-1c*        cagcttactaccgtggtgctatgggcatattgctagtctatgatgttacagatgaagcat    304
                  Forward GTGGWGCYATGGGHATHTTGC

RabD-1          gcagttattatagaggagcacatggaattattattgtttatgatgtcactgacatcgaaa    283
RabD-2c         gcagctactatcgtggggctcatggcataattgttgtttatgatgtcactgaccaagaga    283
RabD-2a         gtagctactaccgtggagcacatggaatcattattgtttatgatgtgacagatgaagaga    283
RabD-2a*        gtagctactatcgtggggcacatggaatcattattgtttatgacgtgacagatgaagaga    283
RabC-2a         gttcttactatagaGgagcacaaggaatcattctcgtttatgatgtaacaagaagagata    406
RabC-1a         gttcatattatagaggtgcacaaggaataattatggtgtacgatgtaacacggcgggaaa    515
RabC-1c         gttcgtactatcgaggagcacaaggaatcatcatggtttatgatgtaacacggagagaaa    295
RabC-1b         gttcttattaccgaggtgcgcaagggatcattatggtttacgatgtcactcggcgagata    292
RabH-1d         caagctacattagggactcatctgttgctgtcattgtgtatgatgttgcaagtcgtcaga    286
RabH-1d*        caagctacatcagggactcgtctgttgctgtcatagtttatgatgttgcaagccgtcaaa    286
RabH-1e         ctagctatatccgagattcttcggtcgcagttattgtatatgatgtagccaaccggcaat    286
RabH-1e*        caagctacataagagattcttctgttgcagttattgtatatgatgtagctaacaggcaat    286
RabG-3f         ttgctttctatcgcggtgctgattgctgtgttcttgtatatgatgttaattcaatgaaat    283
RabG-3d         ttgctttctaccgaggtgcagactgctgtgtccttgtttatgatgtaaatgtcatgaaat    283
RabG-3c         ttgctttctaccgaggtgctgattgttgcgtccttgtttatgacgtgaacgtcatgaaat    109
RabG-3e         tggctttctaccgaggtgctgattgttgcgtccttgtttatgacgtgaacgtcatgaaat    298
RabG-3b*        ctgcattttatagaggggcagattgctgtgttttggtatatgatgtaaatatacacaaaa    289
RabG-3a         ttgcattttatagaggggcggattgctgtgttcttgtgtatgatgtgaacgtcatgaagt    283
RabG-3b         ttgcgttttacagaggagcagattgttgtgttctagtctatgatgttaatgtcatgaagt    283
RabF-1          cactgtattatcgtggtgcagcggttgcagttattgtctacgatataacaagcccagaat    358
RabF-2b         ctatgtat----------------------------------------------------    231
```

```
RabB-1c*      catttgatcacttggataattggttgaaacaaatattggaag---atggtaatgagaaa-    384
RabB-1b       catttaatcatttagcaagttggttggaagatgcccggcagc---atgcaaatcctaac-    333
RabB-1c       catttaatcacttggctagctggttggaagatgcaaggcagc---atgcaaatgcaaat-    333
             Forward TCAYTTRGCWAGYTGGTTGGAAGA

RabA-5a       catttgatagcattggtcgatggctcaacgaacttcacactc---actctgatatgaac-    357
RabA-5b       cttttgatagtatcaagaggtggcttgatgaacttactactc---aaaatgatagcacg-    351
RabA-5e       cttttgatagcgtcggtcgttggctcgatgaactcaagactc---attgcgatacgacg-    357
RabA-3        cgttcgatcatgtagctagatgggttgaagaacttcgatcac---acgctgacacttcc-    375
RabA-3*       catttgatcatgttgctaaatgggttgaggaactaagatcac---attcagataacacc-    387
RabA-4c       cctttaaccatgttgaaaaatggctagacgaactacacatgc---atgccgataaaaac-    360
RabA-4a       gctttgatcacatacctagatggttagaagaactgcgtaacc---atgctgataagaat-    366
RabA-4a*      cctttgatcatataccccgttggctagaagaacttcgtaacc---atgctgacaagaat-    369
RabA-4d       cgttcgatcacatggcaagatggttggaagaattgagaggtc---acgccgacaaaaac-    387
RabA-4d*      catttgatcacatggcaaggtggttagaggaactgaggggtc---atgcagacgccaac-    360
RabA-6a       catttattaatatagaaaaatggttatatgagcttagagagt---ttggaaatgaagac-    351
RabA-6a*      gttatgagagtgtaggaaaatggttagtggagctaagggagt---ttggtggggaagac-    351

RabA-1g       catttgaaaacgtggaaagatggttgaaggagcttcgagatc---acaccgatgcctac-    348
RabA-1g*      ctttcgaaaacatggagagatggttaaaggaacttcgggatc---acacagatgccaac-    354
RabA-1f       catttgaaaatgtggagagatggctaaaagagctgagagatc---acacagatgccaac-    354
RabA-1f*      catttgaaaatgtggagagatggttgaaggagctgagagacc---acacagatgccaac-    354
RabA-1c       catttgagactgctgggagatggttgaaggagttaagggatc---atacagaccccaac-    354
RabA-1d       catttgagaacgctgccaggtggttgaaagagttgagagatc---acacagaccccaac-    354
RabA-1b       catttgagaacgtcgacagatggctgaaagaactgcgaaacc---acacagattcaaac-    354
RabA-1b*      catttgagaatgttgatagatggctgaaagaattaaggaatc---acacagattcaaac-    354
                                  Forward RAARGARYTDMGRRATCACACA

RabA-2a       cattcgacaatgtcagcagatggcttaaggaactgagagatc---acgctgatgctaac-    351
RabA-2d       cctttgacaatgtccaaaggtggctgcgtgaattgagggacc---atgcagattctaac-    351
RabA-2b       catttgataatgttcaaaggtggcttcgtgaactaagagacc---atgcagattccaac-    351
RabA-2b*      catttgaaaatgtacaaaggtggcttcgtgaattaagggatc---atgcagattcgaac-    351
                                                       Forward TTCBAAC

RabE-1a*      catttaacaatatcaaaaattggatgcatagcattga---gcaatatgcttccgacaat-    366
RabE-1a       cgtttaacaacatcaagaattggattcgcaacattga---gcaacatgcttccgataat-    351
RabE-1c       ctttcaacaatattaggaattggattcgcaatattga---acaacatgcttctgacaat-    360
RabE-1c*      catttaacaatatcaggaattggattcgcaacattga---gcaacatgcttcggacaat-    360

RabD-1        gtttcaacaatgttaagcaatggttgcacgaaattga---tagatatgcaaatgacact-    339
RabD-2c       gctttaacaatgttaagcagtggctgaatgaaattga---ccgttatgcaagtgaaaat-    339
RabD-2a       gcttcaataatgtgaagcaatggctcagtgaaattga---ccgctatgccagtgataat-    339
RabD-2a*      gcttcaataatgtgaagcagtggctcagtgaaatcga---ccgctatgccagtgataat-    339
                                    Forward TGAAATTGACCGHTATGCMAGT

RabC-2a       cctttacaaacttaTcagaggtgtggtccaaagaagtggaactttattcaactaatcaga    466
RabC-1a       ctttacaaatctatctgatatatgggctaaagaaattgacttatactcaacaaatcaag    575
RabC-1c       catttacaaatctctctgaagtatgggcaaaggaaatagacctttattcaacaaatcaag    355
RabC-1b       catttacaaatctctctgaagtatgggctaaggaaatagacctctattcaacaaatcagg    352
RabH-1d       cttttctaaacacatcaaagtggattgaagaggtgcgcagtg---agagaggaagtgatg    343
RabH-1d*      ctttccttaacacgtcaaagtggattgaagaggttcgcagtg---agagaggcagtgatg    343
RabH-1e       catttcttaacactaacaggtgggttgaggaagtacgcacag---aacgaggcagcgatg    343
RabH-1e*      catttctgaacactaacaagtgggttgaggaggtacgtcaag---aacgtggcagtgatg    343
RabF-1        ctttcagcaaagcacagtactgggttaaggagctacaaaaac---atggaagccctgata    415
RabF-2b       ------------------------------------------------------------    231

RabG-3f       catttgaaaaccttaacaactggagggaggaatttctgattc---aagcaaatccatccg    340
RabG-3d       cttttgaaaaccttaaccactggcgagaagaattcctcattc---aggccagtccatctg    340
RabG-3c       cttttgaaaaccttaaccattggagagaagaatttctcattc---aggctagtccatctg    166
RabG-3e       cttttgaaaaccttaaccattggagagaagaatttctcattc---aggctagtccatctg    355
RabG-3b*      catttgatacattaaacaattggcatgatgattttcttaaac---agacagatacagaaa    346
RabG-3a       cgtttgattccctcgacaactggcacgaggagtttctcaaac---aggcaaaccctcctg    340
RabG-3b       catttgatacgcttgagaactggcacgaggagtttctcaaac---aggcaaacccttctg    340
                   Forward TTRACMAYTGGMRNGARGART
```

```
RabB-1c*      --atgg---------tagttatgttaatcggaacaagtgcgatct---tatcgata---    427
RabB-1b       --atga---------caatcacgctcatagggaacaagtgcgatct---atctcacc---    376
RabB-1c       --atga---------caattatgctgattggcaacaaatgtgatct---tgctcata---    376

RabA-5a       --gttg---------tcacgatacttgttgggaacaagtcagatct---taaggatg---    400
RabA-5b       --gtag---------caaccatgttggtgggaaataagtgtgattt---ggagaata---    394
RabA-5e       --gtgg---------caatgatgttggtgggaaacaaatgtgattt---ggataata---    400
                                             Forward ATYTKRAKRATRYVAGRGMRGTG

RabA-3        --attg---------tgattatgctaattggtaacaaaggtgatct---tgtggatc---    418
RabA-3*       --attg---------tgatcatgctagttggtaacaaaggtgacct---tgttgact---    430
RabA-4c       --atag---------ttgtcatgctcgttggcaacaagtctgacct---gaattcta---    403
RabA-4a       --atag---------tcataattcttataggaaacaaaagtgatct---tgagaacc---    409
RabA-4a*      --atag---------tcatcattcttgcagggaacaagagtgatct---cgagaacc---    412
RabA-4d       --atcg---------tcatcatgctaattggcaacaaatgtgatct---tgctagtc---    430
RabA-4d*      --attg---------tcataatgctaattggcaataaatgtgattt---gggaactc---    403
RabA-6a       --atgg---------tgataattcttgtagcaaacaaatctgattt---gagtcaat---    394
RabA-6a*      --atgg---------tggttattcttgtgggaaacaaatgtgatct---tagtgaat---    394
RabA-1g       --gtgg---------taatcatgcttgtaggaaacaaagcagatct---tcgacact---    391
RabA-1g*      --attg---------taattatgcttgtggggaacaaggctgatct---tcgtcacc---    397
RabA-1f       --attg---------tcgtgatgcttgtaggtaacaaagcagactt---gcgtcatt---    397
RabA-1f*      --attg---------tggtgatgcttgttgggaacaaagcagactt---gcgtcatt---    397
RabA-1c       --attg---------ttgtcatgcttattggaaataaatcagatct---tagacacc---    397
RabA-1d       --atcg---------tggttatgcttattggaaataagtcggatct---ccgacacc---    397
RabA-1b       --attg---------ttgtgatgcttgttgggaacaagtcagatct---tcgtcatc---    397
RabA-1b*      --attg---------ttgtgatgcttgttgggaacaagtcagatct---tcgacacc---    397

RabA-2a       --attg---------tcatcatgttgattgctaacaaaaccgatct---gaaacatc---    394
RabA-2d       --atag---------tgatcatgatggctggaaataagtctgattt---gaaccatc---    394
RabA-2b       --attg---------ttattatgatggctggaaataaatctgattt---gaatcatc---    394
RabA-2b*      --attg---------ttattatgatggctggaaacaaatctgattt---gaatcatc---    394
          Forward TTCBAACATTGTKATYATGATG

RabE-1a*      --gtca---------acaaaatactggttgggaacaaagctgacatgaatgaaagga---    412
RabE-1a       --gtca---------acaagatattagtggggaacaaggctgacatggacgaaagca---    397
RabE-1c       --gtaa---------acaagatacttgtgggaaacaaggctgatatggatgaaagta---    406
RabE-1c*      --gtta---------acaaaatactggtgggaaacaaagcagacatggatgaaagca---    406
                                                         GAAAGBA

RabD-1        --gtat---------gcaagctacttgtagggaacaaatgtgatctttctgataaca---    385
RabD-2c       --gtaa---------acaagcttctagttggaaacaagtgtgatctcgcggcaaata---    385
RabD-2a       --gtta---------acaagctcttggttggcaacaagtgtgatctgacagcaaata---    385
RabD-2a*      --gtta---------acaagcttttggttggaaacaagagtgatctgacagcaaata---    385
RabC-2a       attgtg---------tgaagatgCtagttggaaataaagttgatag---agactctg---    511
RabC-1a       actgta---------tcaagatgcttgtaggaaacaaagttgacaa---ggaaagtg---    620
RabC-1c       actgcg---------tgaagatgcttgttggaaacaaagtagataa---ggagggtg---    400
RabC-1b       actgca---------tcaagatgcttgttggaaacaaagtggataa---ggagagtg---    397
RabH-1d       ttattg------------ttgttcttgttgggaacaaaactgaccttgtggataaga---    388
RabH-1d*      ttattg------------ttgtgcttgttgggaacaaaactgaccttgttgaaaaga---    388
RabH-1e       ttgtta------------tcgttttggttggaaacaaaactgatcttgttgaaaaaa---    388
RabH-1e*      ttatca------------ttgtattggttggaaacaaaactgatcttgtcgataaaa---    388
RabG-3f       atccagagaattttcctttgttgttataggaacaagatagatattgacggtgggaaca    400
RabG-3d       accctgaaaacttcccatttgtggtgttgggaacaaaatagatgttgatggtgggaata    400
RabG-3c       accctgaaaactttccttttgttgtcttgggaacaaaatcgatgttgatggtggaaata    226
RabG-3e       accctgaaaactttccttttgttgtcttgggaacaaaatcgatgttgatggtggaaata    415
RabG-3b*      atcctgatgcatttccctttgtattactcgggaacaaggttgatgtagatggtggaaaca    406
RabG-3a       atacaaggacatttccgtttatattgcttggaaacaagattgatattgatggtgggaata    400
RabG-3b       atccaaggactttcccatttatattgcttggaaacaaaattgatattgatggcgggaata    400

RabF-1        tagtga------------tggcattagttggtaataaagctgaccttcacgag------a    457
RabF-2b       -----------------------------------------------------------    231
                   CCTGATATAGTGATGGCATTAG Reverse
```

```
RabB-1c*        atcgagttgtgacgaccgaagaaggcgagca---atttgcgagggctaatggtttgattt     484
RabB-1b         gaagggcggtaagcaaagaggagggagagca---gtttgctaaggaaaatggacttttat     433
RabB-1c         ggcgggctgtaagcaccgaggaaggtgagca---gtttgcaaaggagcatgggttgatct     433

RabA-5a         cgagggaggtgcctacaaccgaaggcaaggc---cttagctgaggcacagggtttgttct     457
RabA-5b         tcagagaagtgagcacagaggagggaaaaac---tcttgcagaagaagaaggtttgttct     451
RabA-5e         taagggcagtgagtgttgaagaaggaaaaag---ccttgcggaatcagaaggtttgtttt     457
                YVAGRGMRGTG

RabA-3          aaagagtggtacacaccgaatatgctgtcga---gtttgcagaggatcaaggcctctttt     475
RabA-3*         tgagagttgttcctactgaagatgcaattga---gtttgctgatgaacaaggactattct     487
RabA-4c         ttcgagaagtgccgaccgaggaagcaaaaga---ctttgccaagcagaaaggtctcttct     460
RabA-4a         agcgagcagtaccaacagaggatgcaaaaga---atttgctgagaaagaaggcttatttt     466
RabA-4a*        agcgtgatgtacccacagaggatgcaaaaga---gtttgctgagaaagaaggtttatttt     469
RabA-4d         ttagagcagttccaacggaagacgcagagga---gtttgcacaaagagagaacctttttct     487
RabA-4d*        ttagagcagtaccaactgaagatgcccagga---gtttgcacaaagagagaatctattct     460

RabA-6a         caagacaagttgagaaagaagaaggaaaagg---gtttgctgaaaaagaaagtttatgtt     451
RabA-6a*        caagagaagttgataaagaagagggtaaagc---gtttgcagaggaagaagggttgtgtt     451
                   GASAAGTTGAKAAAGAAGARGG Reverse

RabA-1g         tgcttgctgtgtccacggaagaagccactgc---atttgcagagaaagaaaacatatatt     448
RabA-1g*        tgcgtgccgtgtccactgacgatgctaaggc---atttgctgaaagagaaaatacgtttt     454
RabA-1f         tacgagcagtttcaactgaagatgctacagc---ttttgctgaacggaaaatacatttt     454
RabA-1f*        tgcgagcagtttcaactgaagatgcaacggc---tttcgctgaaagagagaacacatttt     454
RabA-1c         ttgtttctgtctcaactgaagatggaaaagc---ttttgcagagaaagaatccctctact     454
RabA-1d         ttgttgctgtcacaacagaagatggaaaatc---ttttgcagagagggagtctctctact     454
RabA-1b         ttgtagctgtatcaacagaagatgggaagtc---ttacgctgagaaagaatcactttact     454
RabA-1b*        tagtagctgtatcaactgaagacggaaaggc---ttatgctgagaaagaatcactgtact     454
RabA-2a         tccgtgcggttgcaactgaggatgctcagag---ttatgctgagaaggaaggcctttctt     451
RabA-2d         ttagagctgtttcagaggatgatggccaagc---attggctgagaaggaaggtctctctt     451
RabA-2b         taagagcagtatcttctgatgatgctcaaag---tttggctgagaaagagagtctttcat     451
RabA-2b*        taagagcagtttcaactgaagatgctgaaag---tttggctgaaaaggaaggtctttcat     451

RabE-1a*        aaagggccgtgcctacctctaagggccaagc---actagccgacgagtatggaatcaaat     469
RabE-1a         aaagggctgttccaacttcaaagggtcaagc---tctggcagatgaatatggcatcaagt     454
RabE-1c         aaagggctgtacctaccgccaaaggtcaagc---tcttgctgatgaatatggtatcaagt     463
RabE-1c*        aaagggctgtgccaacatccaaaggccaagc---actggctgatgagtatggaatcaaat     463
       GAAAGBAAAAGGGCTGTDCCWA Reverse

RabD-1          agg---ttgttcacacccacactgccaaggc---atttgcagacgagcttggtatccctt     439
RabD-2c         aag---ttgtgtcctctgaaacagcaaaggc---atttgcagatgaaattggaattcctt     439
RabD-2a         gag---ctgtgccatatgaaacagctaaagc---atttgcagatgaaataggcatacctt     439
RabD-2a*        gag---ctgtctcatatgatacagctaaaga---atttgcagatcaaatcggcatacctt     439
                                                                        CCTT

RabC-2a         aaagggttgtgagcacagaagagggtttaGc---tctagccaaagagtttggatgtttgt     568
RabC-1a         aaagggttgttagcaaaaaggaagcggtgga---ctttgcaaggcaatatggatgccttt     677
RabC-1c         atagagttgtgacaaagaaagagggaatgga---cttcgctagggaatatggttgcctct     457
RabC-1b         atagagttgtgacaaagaaagagggaataga---ctttgcaagggaatatggttgcctat     454
RabH-1d         ---ggcaagtctccacagaagaaggggaagc---gaagtctcgggagctaaacgttatgt     442
RabH-1d*        ---ggcaagtctctacagaggaaggggaagc---taaatctcgtgagcttaatgtcatgt     442
RabH-1e         ---ggcaagtctctatagaggaaggagatgc---caagtcgcgtgagagcggtatcatgt     442
RabH-1e*        ---ggcaagtttctatagaggaaggagatgc---caagtcccgagagtttggaatcatgt     442
RabG-3f         gtagagtggtctcagaaaagaaggcccgggcatggtgtgcatcaaaaggaaatatcccat     460
RabG-3d         gccgagttgtttctgagaaaaaagcaaaagcatggtgtgcttccaaggggaacataccgt     460
RabG-3c         gcagagttatttctgagaagaaagcaaaggcatggtgtgcttcaaagggaaacataccat     286
RabG-3e         gcagagttatttctgagaagaaagcaaaggcatggtgtgcttcaaagggaaacataccat     475
RabG-3b*        gtagaagggttactgagaagaaagctagggaatggtgtgcttctaggggtaacataccat     466
RabG-3a         gtcgagtggtttctgagaagaaagcaaaggactggtgtgcttcaaaagggaatattccct     460
RabG-3b         gtcgtgtggtttctgaaaagaaagcgaaggactggtgtacttcaaaagggaatattcctt     460
RabF-1          agcgggaagtggctgttcaggatggcatgga---ctatgcagagaagaatggaatgtttt     514
RabF-2b         ------------------------------------------------------------     231
```

```
RabB-1c*        ttatggagacttcggccaaaactgctaagaatgttgaagaaatatttgtgaaatcagctt    544
RabB-1b         tcatggaggcttctgccaagacagctcaaaatgtagaagaggctttcataaagactgctg    493
RabB-1c         tcatggaggcctcagcaaaaactgctcagaacgttgaagaggcattcataaaaacagctg    493
                                       GCTCARAAYGTWGAAGAGGCWT Reverse

RabA-4c         tcatcgaaacatctgcgcttgactccaacaatgtagaaccagctttccttggtcttcttt    520
RabA-4a         tcttagagacctctgcacttgaagcaactaatgttgagacagccttcacgactgttttga    526
RabA-4a*        tcttagagacgtcggcattggaagcaactaatgttgaagaagccttcatgacagttttga    529
RabA-4d         ttatggagacatcagcgctggaatccaccaacgtcgaaacatgctttctaactatttaa    547
RabA-4d*        ttatggagacatcagcacttgagtctactaatgtggaaactgcctttctgactattctaa    520
                                                                       TYTDA

RabA-1g         tcatggaaacctctgcgcttgagtcattgaatgtggacaatgcttttgttgaagtgctca    508
RabA-1g*        ttatggaaacgtctgcgctcgagtctttaaatgtcgacaattctttcacagaagtgctca    514
RabA-1f         tcatggagacatctgcccttgagtcattgaacgtagaaagtgcgttcactgaagtgttga    514
RabA-1f*        tcatggaaacttctgccctcgagtccatgaatgtcgaaaatgccttcactgaagtgctga    514
RabA-1c         tcatggagacttctgctttagaagcaaccaatgttgaaaatgctttcaccgaggttctta    514
RabA-1d         tcatggagacttctgctttggaagcaaccaatgtagaaaatgcattcactgaggttcttt    514
RabA-1b         tcatggaaacctcagctcttgaggcttctaacgtagagaatgcatttgctgaagttctca    514
RabA-1b*        tcatggaaacctcggctttggaagcaacaaatgtagagaatgcattcgccgaagttttga    514
                                                        TGCNTTYRCYGARGTKYBBACK

RabA-5a         ttatggagacatcggctctcgattcatcgaacgtagtagctgcttttcaaacagttgtaa    517
RabA-5b         ttatggagacatcagcactcgaatctaccaatgttcaaacggcttttgagattgttatcc    511
RabA-5e         tcatggaaacttctgctttggactctacaaatgtgaaaacagcttttgaaatggttatta    517
RabA-3          tctctgagacttcagcttttactggtgaaaatgtcaactctgcatttttaaattgcttg    535
RabA-3*         tttatgagacttcagctctcactggtgagaatgttgagactgcatttatcaaattgtttg    547
RabA-6a         tcatggaaacttctgctttgcataatttaaatgttgaggatgcattcttagaaatgatta    511
RabA-6a*        ttatggaaacctctgctttgaagaatctgaatgttgaacaagtgtttttacaaatgatca    511
RabA-2a         tcattgagacatctgctcttcaagctaccaatgtggagaaggctttccagactatccttg    511
RabA-2d         ttcttgagacatctgcactggaagcaaccaacattgagaaggcattccaaaccatattga    511
RabA-2b         ttctagagacttcagcattggaagctttaaatgttgagaaggcttttcaaaccattttgt    511
RabA-2b*        ttctagagacatcagcattggaagcatataatgttgagaaagcatttcaaaccatattgt    511
RabE-1a*        ttttcgaaacaagtgcaaagacaaatttaaatgtggacaaagttttcttctcgattgtga    529
RabE-1a         tttttgaaactagtgcaaaaactaatatgaatgtggatgaggttttcttttcaatagctc    514
RabE-1c         tctttgagactagtgcaaaaacgaacatgaatgtggaggaagttttcttttcaatagcaa    523
RabE-1c*        tctttgaaactagtgcaaaaacaaatctaaatgtggaggaagttttcttctccatagcaa    523

RabD-1          tccttgagacaagtgctaaagactcagtcaatgtggagcaggctttcttgactatggcag    499
RabD-2c         tcatggaaactagtgctaaaaatgccaccaacgtggaacaggctttcatggccatggctg    499
RabD-2a         ttatggagacaagtgcaaaagattctacaaatgtcgaacaggcctttatggcaatggctt    499
RabD-2a*        ttatggaaacaagtgcaaaagatgctacaaatgtggaaggggcattcatggccatggctg    499
            CCTTTYATGGARACAAGTGCWA Reverse

RabC-2a         tttttgaatgcagtgccaaaactcgagaaaatGtagacaagtgtttcgaagaacttgcac    628
RabC-1a         atactgaatgcagtgcgaaaacccgagtaaatgttgagcagtgttttgaagaacttgtag    737
RabC-1c         ttattgaatgcagtgctaaaactcgagtaaatgtgcagcaatgctttgaagagcttgttt    517
RabC-1b         ttactgaatgcagtgctaaaactcgagttaacgtacaacaatgctttgaagagcttgttt    514
RabH-1d         ttattgaagctagtgccaaagccggctttaatataaaagctctctttcgaaaaattgctg    502
RabH-1d*        tcattgaagctagtgcaaaagccggctttaatataaaggctctctttcgaaaaattgctg    502
RabH-1e         ttatagaaaccagtgcaaaagcaggatttaatatcaagcctttatttcgcaagattgctg    502
RabH-1e*        ttatagaaaccagtgcaaaagcaggcttcaatatcaagcctttgtttcgtaagattgctt    502

RabG-3f         attatgagacatctgccaaagagggtattaatgttgaagaagcattccaatgcatagcaa    520
RabG-3d         actttgagacctctgcaaaggagggatttaatgttgaagctgcttttgagtgtataacca    520
RabG-3c         actttgagacatctgcaaaagaaggatttaatgttgaagctgctttccagtgtatagcaa    346
RabG-3e         actttgagacatctgcaaaagaaggatttaatgttgaagctgctttccagtgtatagcaa    535
RabG-3b*        atttttgagacctctgcaaaagagggatacaatgttgatgatgcatttttatgtgttgcta    526
RabG-3a         actttgagacatctgcaaaagaggattacaatgttgatgctgcattcctgtgtattgcaa    520
RabG-3b         actttgagacatctgcaaaagaggactacaatgttgatgctgcattcctttgtattgcca    520
                   TTGAGACMTCTGCAAAAGARGR Reverse

RabF-1          tcatagagacatctgcaaagacagcggataatataaatgaactgtttgaggaaattgcca    574
RabF-2b         ------------------------------------------------------------    231
```

```
RabB-1c*      caactatatgtaataagattaaggatggtgtttttgatgtaaagaatgataattgtggga    604
RabB-1b       caaagattcttcagaacatccaggatggtgtcttcgatgtgtccaatgagacatgtggta    553
RabB-1c       caaccatatacaaaaagattcaagatggactttttgatgtatcaaatgagtcttatggaa    553
RabA-6a       ctaagatacatgatatcataagccaaaaaagtttggaagaaaaaattaatgggccaaagt    571
RabA-6a*      caaagatacttgagatcacaagtcagagaagtttggaagctaaaatggatgagaaaccaa    571

RabA-5a       aagagatctacaacatattaagccggaaggttatgatgtctcaagagctcaataaacagg    577
RabA-5b       gtgaaatttacaataacatcagccgcaaagtcttaaactctgattcttataaggctgaat    571
RabA-5e       gagagatatacaacaatgtcagcagaaaggtcctcaactcagacacttacaaggctgaat    577
                         AAYAWHDTMAGCMGVAARGTYH Reverse

RabA-3        aagagattaataaggttgtttcaaaaaggtcgttggactgtaataatggaaggccaatg     595
RabA-3*       aagagattaatagggtggtttgtaagaaggcattggaatgtggtaatggttactatgatg    607
      AATTGYTTGAAGAGATTAATAR Reverse

RabA-4c       cacaaatatatatggcagtcagtaagaagcacatcactgcagatggacatgaatcaaatt    580
RabA-4a       cagaaatatttaacattgtcaataagaagaacctggctgctgatgagaatcaggaaaatg    586
RabA-4a*      cagaaatatacaacattgtcaacaagaagaccctagctgctgatgaaaatcatggaaatg    589
RabA-4d       ccgagatataccgaatcaatgccaagaaaacactcacttcaaatggtgatgcagatcata    607
RabA-4d*      ccgaaatataccgacttatcagcaagaaaacactcactgccaacgatgacgcagatcctg    580
          TYTDACMGAAATATAYMDVVTHR Reverse

RabA-1g       ctcaaatatacaatgttgtgagtaggaagacacttgagaaaacgaatgactctgcatctt    568
RabA-1g*      ctcaaatatatcgagttgttagtaggaagactctcgagattggagatgaccctgctgcct    574
RabA-1f       cgcagatctatcgtgttgtaagtaagaaggcccttgagatcggtgacgatccagctgctt    574
RabA-1f*      cgcagatctaccgtgttgtgagcaagaaagcccttgagattggtgatgatccagcagcac    574
RabA-1c       ctcagatataccacatagtgagcaagagatctgttgaggctggtgatggcgctagtgctt    574
RabA-1d       ctcagatataccgcatactaagcaagaaagcagtcgaggctggcgaaagtggaagctcct    574
RabA-1b       ctcagatctaccgtattgttagcaagaaagctgtagagggtgctgaaaatggaaacgcgt    574
RabA-1b*      ctcaaatctaccatttgttagcaaaaagacagttgacagcgcagaaaatggaactgcaa     574
              CK Reverse

RabA-2a       gagagatataccgcattatcagtaagaaatcactttcttccaatcaacctcgtgataatc    571
RabA-2d       cagagatttatcatattgtaagcaaaaaagcacttgcagctcaggaagcagcttctggta    571
RabA-2b       ttgatatttatcaaattataagtaaaaaggcacttgctgcacaagagtctgcttctactc    571
RabA-2b*      ttgatatatatcacattataagtaaaaaagcacttgcagcacaagaagctacttctacta    571
                                  GTAAAAAAGCACTTGCWGCHCA Reverse

RabE-1a*      aagatataaaacgaaggcttgatgacattgactcgaagacaccaccgataggaattagaa    589
RabE-1a       gggacatcaaacaaagacttgcagaaagtgactcaaaggctgagccccagtcactcaaga    574
RabE-1c       gagacatcaagcaaaggcttgcagacacagattctagagccgagccccagactatcaaga    583
RabE-1c*      gagacataaaacaaaggcttgccgacactgataacaaggcagagccaacaaccatcaaaa    583
RabD-1        cagagattaagaagaagatgggaaaccaaccagctagtagtaagtcagcgga---atctg    556
RabD-2c       ctgaaattaaaaacagaatggcaagtcaaccag---cgaacaatgccaggcctccaacag    556
RabD-2a       cttccatcaaggatagaatggcaagccaaccta---caaacaatgcaaggcctccaaccg    556
RabD-2a*      ctgccatcaaggatagaatggcaagccaaccgtccgcaaacaatgcaaggcctccgacgg    559
RabC-2a       taaagataatggaagttcctagtcttttggaaGaaggatcaacatcagtgaaaaggaata    688
RabC-1a       tgaagatttggagacaccaagcctcttggctgagggctcatctggtgtgaaaaaaaaca     797
RabC-1c       tgaagactcttgatacacctagcctcttagctgagggctctaagggagtaaaaaataaca    577
RabC-1b       tgaaggttttggacacacctagcctcttagccgagggatctaaaggggtcaaaaagaaca    574
RabH-1d       ctgcattacctggaatggaaacactatctacaacaaaacaagaagacatggtcgatgtga    562
RabH-1d*      ctgcattacctggaatggaaaccctatcttccacaaaacaagaagatctggttgatgtga    562
RabH-1e       cagctttgcccgggatggaaactctttcttcaacaaaacaagaagacatggttgatgtaa    562
RabH-1e*      ctgccctgccagggatggaaactctttcttccacaaagcaggaagacatggttgacgtaa    562
RabG-3f       agaatgccctgaaaagtggggaaga-------------------ggaagaactatacctgc    562
RabG-3d       aaaatgctctcaagaatgaacctga-------------------agaagaattatacatgc    562
RabG-3c       aaaatgcactcaagaatgaacctga-------------------agaagaaatgtatctgc    388
RabG-3e       aaaatgcactcaagaatgaacctga-------------------agaagaaatgtatctgc    577
RabG-3b*      aagttgcatcacaaaatgaacgtga-------------------tctagatatttatttcc    568
RabG-3a       agactgccctggccaatgagcgtga-------------------acaggacatatattttc    562
RabG-3b       agactgctttagccaatgagcgtga-------------------ccaggacatatattttc    562
RabF-1        aaagactgcctc------------------------------------------------    586
RabF-2b       ------------------------------------------------------------    231
```

```
RabB-1c*     ttagagtaggatatggtggagg---taggtgtgatgaattttaga--------------   647
RabB-1b      taaaagttgggtatggacgtcc---acaaggccagtcaggaggcagagatggaactgttg   610
RabB-1c      taaaagtaggatatggtggaat---tcctggaccatctggaggtagggatggcccttctg   610
RabA-5a      atccttcctggattgaaaatgg---aaagactgttgttctacaaga---aggggataaag   631
RabA-5b      tgtctgt---------gaatcg---agtaagcctggttaatggggc---aggatcaaaga   616
RabA-5e      tatctgt---------caacag---ggttagtcttgttaacgatgg---caactctacat   622
RabA-3       ---ctattattca---taaagg---attggaaatt------gatat---aatctcacctc   637
RabA-3*      ctgcttctacaat---taaagg---ttcaaagatt------gatgt---aatctcaggtg   652
RabA-4c      gggataaagtaaaccttgaatt---tgtgggaacaaaa---attaa---ggtctcatcac   631
RabA-4a      gcaactcgacatc---tctagc---tggcaagaag------attat---tgttcctggtc   631
RabA-4a*     gaaactcggcatc---tttggc---tagccagaaa------cttat---tattccaggcc   634
RabA-4d      gtgatacttcaggtcttcttaa---aggaagtaga------ataat---tgttcctggta   655
RabA-4d*     ctggtagttcagggcttctgaa---aggaaccaaa------ataat---tgttcctaacc   628
RabA-6a      taagtgttcctaatggaaagga---aattcatatagtt---gatga---agtgactgcca   622
RabA-6a*     ttagtctttggaatgggaagga---gattagtttattggccgatga---agtcacagcta   625
RabA-1g      tgtctaagggagagacca---t---caatattggtagtaaagatga---tgtttctgagt   619
RabA-1g*     tgcccaaaggacagacaa---t---caatgttggcagtagagatga---tgtttcagctg   625
RabA-1f      tacctaaaggacagacga---t---tagtgttgggtcccgtgatga---tgtatcagctg   625
RabA-1f*     taccaaaaggacagacaa---t---tgatgttggatctcgggatga---tgtatcggctg   625
RabA-1c      caggtgttccatctaaaggaca---aacaataaatgtgaaagagga---ttcttcagttt   628
RabA-1d      cagcagtcccatctaaaggaca---gacaataaatgtgaaagagga---ttcttcagttt   628
RabA-1b      ---ctgtgccagccaaaggaga---gaaaatagatcttaaaaatga---tgtttctgccc   625
RabA-1b*     ---ctgtgccaaccaaaggaga---gaaaatagatcttaagaatga---tgtgtctgcat   625
RabA-2a      atgatgatgatgcacc---ttc---taatattaatatcaaacaagg---caagaccatca   622
RabA-2d      cctcagttcctggccaaggaac---cacaatcaatgttgcagatac---ctccggcatta   625
RabA-2b      ctagtcttcctcatgg---aac---taccattaatgtgtcaaatat---gtctggtactg   622
RabA-2b*     ctggacttcctcatgg---aac---tactataaatgtttcaaatat---gtctgataata   622
RabE-1a*     tcaacagacaaaatcaggcaaacggagcgtgga---atgcacagaagttggcgtgctgtg   646
RabE-1a      ttaaccaatcagaccagggagctgggtctgctcaagccggacaaacatctgcttgctgtg   634
RabE-1c      ttaaccaacaagattcggcagccgatggtggtcaagctgcacaaaaatcagcttgctgcg   643
RabE-1c*     tcaaccaaga---ctctgcagtggggggctggacaagctgcgcaaaaatctgcttgctgtg   640
RabD-1       ttcagatgaa---------------ggggcaaccaatcccacagaagagcaactgttgtg   601
RabD-2c      tccaaattcg---------------aggacagccaatgaaccaaaagtctggttgctgct   601
RabD-2a      tgcagataag---------------aggacagccagttgggcagaaaggtggctgctgct   601
RabD-2a*     tgcagattag---------------aggacaaccagttgggcaaaaaggtggttgctgct   604
RabC-2a      ttttaaagcaaaaaca---------ggaacctcaagcatcaCaagatggtggttgttgct   739
RabC-1a      tcttcaagcagaaacc---------acaagagtctgatgcatcaactagtagctgttgct   848
RabC-1c      tttttaaggacaagcc---------acctcagtctgatgcatccacaggtggttgttgct   628
RabC-1b      tttttaaggacaagcc---------accccagtctgatgcagccacaagtggttgttgct   625
RabH-1d      acctgaggtcttctgcca---g---ccatgactctcaacctcagtc---tggtggttgtt   613
RabH-1d*     acctaaaatcttctggtg---g---ccatgattctcaaactcagtc---tggtggatgtt   613
RabH-1e      atttaaaatccaccacga---a---ttcatcccagacagagcagca---aggtggaggtt   613
RabH-1e*     atttaaaacctacggtga---a---ttcatcccagacagagcagca---gggaggaggtt   613
RabG-3f      cagacacaattgatgttg---g---aaacagcaatcaacaacgggc---aac---aggat   610
RabG-3d      ctgaagcaattgatgttg---g---cggtggtggacagcaacagag---gtcaacagggt   613
RabG-3c      ctgacacaatcgatgtcg---g---aagtggtggccgacaacagag---atcgactggct   439
RabG-3e      ctgacacaatcgatgtcg---g---aagtggtggccgacaacagag---atcgactggct   628
RabG-3b*     gtggaatatctgaaactc---c---ttcagaaccagaacaacaaag---tggttgtgcat   619
RabG-3a      aagggattcccgaggcgg---t---tccagagaatgagcagagagg---tggtggatgtg   613
RabG-3b      aacctattccagaggctg---c---tccagagaatgagcagagggg---tggatgtgcat   613
RabF-1       -------------------------------------------------------gccca   591
RabF-2b      ------------------------------------------------------------   231
```

```
RabB-1c*      -tttgggaggaggttg------------------------ctgcaaaacttga-------     675
RabB-1b       ctgcaggaggtgcatg------------------------ttgcgg---ctga-------     636
RabB-1c       ctgcaggcagaggctg------------------------ctgcaa---ttga-------     636
RabA-5a       aaaa---agaagcaga------agctgagacaaaaaagggttgttgttcatcttag----     678
RabA-5b       aaa----atctg-----------ct------caatttttcctgttgttcatga-------     648
RabA-5e       cca----agaaa-----------aacaatagctatttttcttgctgttcctga-------     660
RabA-3        ctgaattagaaattac------tgaattcaagaaattaccttcatgttcttgttga----     687
RabA-3*       ctgaattagaaattac------tgagatcaagaaattgtcttcttgctcttgttaa----     702
RabA-4c       aagaaccagaatttcaaaaatctaaaaggagatacaattgttgcagcgttttatag----     687
RabA-4a       ctgcacaagaaatccc------taagaagagcatgtgttgtcagtaa-------------     672
RabA-4a*      ctgcacaggaaatccc------tgctaagagcaacatgtgttgccaggcatcatga----     684
RabA-4d       atcaaatggatgctac------tggaaagaagggtggttgttgttttgcctcatag----     705
RabA-4d*      aagatgtgaatgctct------tgaaaagaagggtggctgctgtggataa----------     672
RabA-6a       ctaaacaagttaattgttgttcaagatga-------------------------------     651
RabA-6a*      ctaaacaggctccttgttgttcaagatga-------------------------------     654
RabA-1g       ttaagaagagtggatgctgcagcactgcatag----------------------------     651
RabA-1g*      ttaaaaaggttggatgctgttcggcgtag-------------------------------     654
RabA-1f       tgaagaaagctggatgttgttctgcttga-------------------------------     654
RabA-1f*      tgaagaaatctggatgttgctctgcttga-------------------------------     654
RabA-1c       tgaaaagatatggatgctgctcaaattag-------------------------------     657
RabA-1d       tgaagagatttggatgctgctcaaactag-------------------------------     657
RabA-1b       tgaagagagttggttgttgttcaagctaa-------------------------------     654
RabA-1b*      tgaagagagttggttgctgctcaagctaa-------------------------------     654
RabA-2a       ccgttggtggaccccaaccctccaatacaaacaactcctgctgtacatcttcaatgtaa-     681
RabA-2d       caaagagagggtgttgt---tccacttaa-------------------------------     651
RabA-2b       tggaaaagaaatcttgttgctcaaattaa-------------------------------     651
RabA-2b*      ctggaaatagaacttgttgctcaaattaa-------------------------------     651
RabE-1a*      gttaa-------------------------------------------------------     651
RabE-1a       gttcttag----------------------------------------------------     642
RabE-1c       gttaa-------------------------------------------------------     648
RabE-1c*      gttga-------------------------------------------------------     645
RabD-1        gctag-------------------------------------------------------     606
RabD-2c       cgacttag----------------------------------------------------     609
RabD-2a       catcttaa----------------------------------------------------     609
RabD-2a*      cttcttag----------------------------------------------------     612
RabC-2a       cttaaaatgctgcaatttgaacattgagttttaactaccacAtattgaactggtcctct-     798
RabC-1a       catggtgattatgatgtatttaaagagctatcaccttccacttttcagtatgatgttaa-     907
RabC-1c       gattttaagaagctacttcacttggaagctgcattttaagaatcacggcctccaagagaa     688
RabC-1b       aagaaaaggctatttcactcaaagtaaaaaatcactgcctattgaagatctcaaaaaaaa     685
RabH-1d       cttgctga----------------------------------------------------     621
RabH-1d*      cttgctga----------------------------------------------------     621
RabH-1e       gctcatgctaa-------------------------------------------------     624
RabH-1e*      gctcatgttag-------------------------------------------------     624
RabG-3f       gtgagtgctga-------------------------------------------------     621
RabG-3d       gtgaatgttga-------------------------------------------------     624
RabG-3c       gtgaatgttga-------------------------------------------------     450
RabG-3e       gtgaatgttga-------------------------------------------------     639
RabG-3b*      gctaa-------------------------------------------------------     624
RabG-3a       catgctga----------------------------------------------------     621
RabG-3b       gctga-------------------------------------------------------     618
RabF-1        gtaattactta-------------------------------------------------     603
RabF-2b       ------------------------------------------------------------     231
```

## Sequences of *CaRab* genes used for the alignment

**>*RabA-1b***
atggcaggttacagagcagacgatgactacgactatctattcaaggtagttctgatcggc
gactccggtgtcggcaaatcgaatcttctctcgcgtttcacgaagaacgagttcaacctt
gagtccaaatccaccattggtgttgagttcgctactcgtactttgaatgttgattctaaa
gtcatcaaggctcagatctgggatactgctggtcaagagaggtatcgtgccatcaccagt
gcttactatcgcggggcagtcggtgcacttcttgtatatgacgtcacacgccatgcaaca
tttgagaacgtcgacagatggctgaaagaactgcgaaaccacacagattcaaacattgtt
gtgatgcttgttgggaacaagtcagatcttcgtcatcttgtagctgtatcaacagaagat
gggaagtcttacgctgagaaagaatcactttacttcatggaaacctcagctcttgaggct
tctaacgtagagaatgcatttgctgaagttctcactcagatctaccgtattgttagcaag
aaagctgtagagggtgctgaaaatggaaacgcgtctgtgccagccaaaggagagaaaata
gatcttaaaaatgatgtttctgccctgaagagagttggttgttgttcaagctaa

**>*RabA-1b****
atgggggatacagagcggatgacgactacgactaccttttcaaagtagttctaatcggt
gattccggcgtagggaagtcaaaccttctttcaaggttcaccaagaacgagttcaacctc
gaatctaagtccaccattggcgtcgagttcgccacacgcacccttaacgtcgacaccaaa
gtcgttaaatctcagatctgggacaccgccggtcaggaaaggtatcgagcgatcaccagt
gcttattatcgtggggctgtaggtgcacttcttgtatacgatgtcacacgccatgcaaca
tttgagaatgttgatagatggctgaaagaattaaggaatcacacagattcaaacattgtt
gtgatgcttgttgggaacaagtcagatcttcgacacctagtagctgtatcaactgaagac
ggaaaggcttatgctgagaaagaatcactgtacttcatggaaacctcggctttggaagca
acaaatgtagagaatgcattcgccgaagttttgactcaaatctaccattttgttagcaaa
aagacagttgacagcgcagaaaatggaactgcaactgtgccaaccaaaggagagaaaata
gatcttaagaatgatgtgtctgcattgaagagagttggttgctgctcaagctaa

**>*RabA-1c***
atgagcgggtacaaagcagatgatgaatacgattacctttttcaagctggttttgattggt
gattctggagttggcaaatctaaccttctttccaggttcaccaaaaacgagttcaatttg
gagtccaagtccaccataggtgtcgaattcgccaccaaaactttgaatattcatgctaaa
atcatcaaggctcagatttgggacactgctggacaagaaaggtaccgtgccattaccagt
gcttactaccgaggagctgtcggggccttacttgtctacgatgtaacccgcagttcaaca
tttgagactgctgggagatggttgaaggagttaagggatcatacagaccccaacattgtt
gtcatgcttattggaaataaatcagatcttagacaccttgtttctgtctcaactgaagat
ggaaaagcttttgcagagaaagaatccctctacttcatggagacttctgctttagaagca
accaatgttgaaaatgctttcaccgaggttcttactcagatataccacatagtgagcaag
agatctgttgaggctggtgatggcgctagtgcttcaggtgttccatctaaaggacaaaca
ataaatgtgaaagaggattcttcagttttgaaaagatatggatgctgctcaaattag

**>*RabA-1d***
atggctgggtacagagcagatgatgagtacgattacttgttcaaacttgttctgattggc
gattccggtgttggtaaatccaatttgctttctaggttcactaggaatgagttcaatttg
gaatctaaatccaccataggtgttgagtttgctaccaagagtttgactattgattccaaa
gttatcaaggctcagatttgggatactgctggtcaggaaaggtaccgtgccattactagt
gcctactatcgaggagctgttggtgccttacttgtttatgatgtcacacggcgggctaca
tttgagaacgctgccaggtggttgaaagagttgagagatcacacagaccccaacatcgtg
gttatgcttattggaaataagtcggatctccgacaccttgttgctgtcacaacagaagat
ggaaaatcttttgcagagagggagtctctctacttcatggagacttctgctttggaagca

accaatgtagaaaatgcattcactgaggttctttctcagatataccgcatactaagcaag
aaagcagtcgaggctggcgaaagtggaagctcctcagcagtcccatctaaaggacagaca
ataaatgtgaaagaggattcttcagttttgaagagatttggatgctgctcaaactag

***>RabA-1f***
atgggggcatacagagccgatgacgattacgattatctattcaaagtggttttgatcgga
gactcaggtgttggtaaatcaaatcttctttcgaggttcacaaagaatgaattcagcctc
gaatctaaatccaccattggcgttgagttcgctaccagaagcattcgtgtagatgataag
gttgtcaaggctcagatttgggatactgccggtcaagaaaggtaccgagcaattacaagt
gcttattatcgtggagctgttggcgctttattagtctatgatgttactcgtcgtgttaca
tttgaaaatgtggagagatggctaaaagagctgagagatcacacagatgccaacattgtc
gtgatgcttgtaggtaacaaagcagacttgcgtcatttacgagcagtttcaactgaagat
gctacagcttttgctgaacgggaaaatacattttcatggagacatctgcccttgagtca
ttgaacgtagaaagtgcgttcactgaagtgttgacgcagatctatcgtgttgtaagtaag
aaggcccttgagatcggtgacgatccagctgctttacctaaaggacagacgattagtgtt
gggtcccgtgatgatgtatcagctgtgaagaaagctggatgttgttctgcttga

***>RabA-1f****
atgggggcatacagagctgatgatgattatgattatctgttcaaggttgttctgattggt
gattctggtgttggaaaatccaacctttgtctagattcaccaagaatgaatttagcctt
gaatcaaaatccaccattggtgttgaatttgcaaccagaagcattagggttgatgataag
gttgtcaaggctcagatttgggatactgctggccaggaaaggtaccgagcaattacaagt
gcatattatagaggagctgttggtgctttactagtgtatgatgttacacgccatgttaca
tttgaaaatgtggagagatggttgaaggagctgagagaccacacagatgccaacattgtg
gtgatgcttgttgggaacaaagcagacttgcgtcatttgcgagcagtttcaactgaagat
gcaacggctttcgctgaaagagagaacacattttcatggaaacttctgccctcgagtcc
atgaatgtcgaaaatgccttcactgaagtgctgacgcagatctaccgtgttgtgagcaag
aaagcccttgagattggtgatgatccagcagcactaccaaaaggacagacaattgatgtt
ggatctcgggatgatgtatcggctgtgaagaaatctggatgttgctctgcttga

***>RabA-1g***
atgggtgccgccgacgaagaatatgattacttgttcaagttggttttgatcggtgactcc
ggtgttggaaagtccaacctttgtctagattcacccgaaatgaattcaacttggaatcc
aaatccaccatcggcgttgaattcgctactcgcagtgttcgtattcttgacaaacttgtc
aaagcccagatttgggacactgctggtcaagaaagatatcgtgcaatcacaagtgcttac
taccgtggagccgttggtgcactgcttgtgtacgatactacaaggcacgtgacatttgaa
aacgtggaaagatggttgaaggagcttcgagatcacaccgatgcctacgtggtaatcatg
cttgtaggaaacaaagcagatcttcgacacttgcttgctgtgtccacggaagaagccact
gcatttgcagagaaagaaaacatatatttcatggaaacctctgcgcttgagtcattgaat
gtggacaatgcttttgttgaagtgctcactcaaatatacaatgttgtgagtaggaagaca
cttgagaaaacgaatgactctgcatctttgtctaagggagagaccatcaatattggtagt
aaagatgatgtttctgagtttaagaagagtggatgctgcagcactgcatag

***>RabA-1g****
atgggtgcgtacagggccgacgacgactacgactacctatttaaggtggttctgatcgga
gactccggcgtcggtaaatccaacctcttgtcacgattcaccaaaaatgaattcagtttg
gaatctaaatccaccattggtgttgaattcgcaacacgcagcatacacgtcgatgataag
attataaaggcacaaatttgggataccgcaggacaagaaagatatcgagcgatcacaagt
gcatattatcgaggcgctgtcggtgcattgcttgtgtatgatgttacaaggcatgtaact
ttcgaaaacatggagagatggttaaaggaacttcgggatcacacagatgccaacattgta
attatgcttgtggggaacaaggctgatcttcgtcacctgcgtgccgtgtccactgacgat
gctaaggcatttgctgaaagagaaaatacgtttttatggaaacgtctgcgctcgagtct

ttaaatgtcgacaattctttcacagaagtgctcactcaaatatatcgagttgttagtagg
aagactctcgagattggagatgaccctgctgccttgcccaaaggacagacaatcaatgtt
ggcagtagagatgatgtttcagctgttaaaaaggttggatgctgttcggcgtag

***>RabA-2a***

atgagtaggagaggagaagaagaatacgattatctgttcaaggtagtactgatcggcgat
tctggcgttggtaaatccaatcttctctcccgattcactcgcaatgagttttgtctcgag
tccaagtccactatcggcgttgaattcgccacccgtaccctcaaggtcgagtcaaaaacc
gttaaagctcagatatgggacaccgccggacaggaacgttacagagcgatcaccagcgcc
tactatcgcggtgctcttggtgctcttcttgtctacgatgtgaccaaaccaattacattc
gacaatgtcagcagatggcttaaggaactgagagatcacgctgatgctaacattgtcatc
atgttgattgctaacaaaaccgatctgaaacatctccgtgcggttgcaactgaggatgct
cagagttatgctgagaaggaaggcctttctttcattgagacatctgctcttcaagctacc
aatgtggagaaggctttccagactatccttggagagatataccgcattatcagtaagaaa
tcactttcttccaatcaacctcgtgataatcatgatgatgatgcaccttctaatattaat
atcaaacaaggcaagaccatcaccgttggtggaccccaaccctccaatacaaacaactcc
tgctgtacatcttcaatgtaa

***>RabA-2b***

atggcatacaaagtagaccatgaatatgactatctcttcaagatcgtactcattggtgat
tctggtgttggaaaatcaaatatactctccaggtttacccgaaatgagttctgtttagaa
tctaaatctaccattggtgttgaattcgctaccagaacaatacaggtggaagggaaaaca
gtgaaagcacaaatatgggacactgcaggtcaagagaggtacagagcaatcacaagtgca
tattacagaggagctgttggtgccttattagtgtacgacataatcaagagacaaacattt
gataatgttcaaaggtggcttcgtgaactaagagaccatgcagattccaacattgttatt
atgatggctggaaataaatctgatttgaatcatctaagagcagtatcttctgatgatgct
caaagtttggctgagaaagagagtctttcatttctagagacttcagcattggaagcttta
aatgttgagaaggcttttcaaaccatttgtttgatatttatcaaattataagtaaaaag
gcacttgctgcacaagagtctgcttctactcctagtcttcctcatggaactaccattaat
gtgtcaaatatgtctggtactgtggaaaagaaatcttgttgctcaaattaa

***>RabA-2b****

atggcatacaaaatagatcatgaatacgattatctattcaaggttgttttaattggggac
tctggtgttggaaaatccaatatcctttctcgttttacaagaaatgagttttgtttggaa
tctaaatctaccattggtgttgaatttgcaacaaggacattacaggtagagggaaagaca
gtaaaggcacaaatatgggacactgctggtcaagaaaggtacagagcaattacaagtgct
tactatagaggagctgttggtgcattattggtttatgacataacaaagagacaaacattt
gaaaatgtacaaaggtggcttcgtgaattaagggatcatgcagattcgaacattgttatt
atgatggctggaaacaaatctgatttgaatcatctaagagcagtttcaactgaagatgct
gaaagtttggctgaaaaggaaggtctttcatttctagagacatcagcattggaagcatat
aatgttgagaaagcatttcaaaccatattgtttgatatatatcacattataagtaaaaaa
gcacttgcagcacaagaagctacttctactactggacttcctcatggaactactataaat
gtttcaaatatgtctgataatactggaaatagaacttgttgctcaaattaa

***>RabA-2d***

atggcgcatagagtggatcatgagtatgattacttgttcaagatcgttttgatcggagat
tctggtgttggaaaatctaacatcctttccaggtttactcgaaacgagttttgtttggag
tccaaatctactattggagttgaattcgccaccagaacccttcaggtagagggaaagact
gtaaaggcacagatatgggacacagcaggtcaggaacggtaccgtgctattaccagtgcc
tattatagaggagctgtgggtgctctccttgtatatgacataactaagaggcaaaccttt
gacaatgtccaaaggtggctgcgtgaattgagggaccatgcagattctaacatagtgatc
atgatggctggaaataagtctgatttgaaccatcttagagctgtttcagaggatgatggc

caagcattggctgagaaggaaggtctctcttttcttgagacatctgcactggaagcaacc
aacattgagaaggcattccaaaccatattgacagagatttatcatattgtaagcaaaaaa
gcacttgcagctcaggaagcagcttctggtacctcagttcctggccaaggaaccacaatc
aatgttgcagatacctccggcattacaaagagagggtgttgttccacttaa

***>RabA-3***

atgaatcaggaaatgaatggggtggaagttgaaagggtagaagagaaaatagattatgtg
tttaaggttgtggtgataggagactcagcagtaggaaagactcaaatactgtcaaggttt
gcaaagaatgagttctgctttgactcaaaatcaaccattggagttgaatttcaaactaaa
actgtcacaataaatggtaaactcatcaaagcacagatctgggatactgctggccaagaa
aggtacagagcagtgacaagtgcatactacagaggagcattaggggcaatgctagtgtac
gacataactaaaagacaaacgttcgatcatgtagctagatgggttgaagaacttcgatca
cacgctgacacttccattgtgattatgctaattggtaacaaaggtgatcttgtggatcaa
agagtggtacacaccgaatatgctgtcgagtttgcagaggatcaaggcctcttttctct
gagacttcagcttttactggtgaaaatgtcaactctgcatttttaaattgcttgaagag
attaataaggttgtttcaaaaaggtcgttggactgtaataatgggaaggccaatgctatt
attcataaaggattggaaattgatataatctcacctcctgaattagaaattactgaattc
aagaaattaccttcatgttcttgttga

***>RabA-3****

atgatccatgatggtgagaatggaaatgagaaaatagattattatgatatggaggagaaa
atagattatgtgtttaaggtgattgtaattggggattcagcagtaggaaagacacaaata
ctgtcaaggttttcaaagaatgagttctgttttgactctaagtcaactattggagttgaa
tttcaaactaggactgttattattaatggtaaagttatcaaagctcagatctgggatact
gctggccaagaaaggtacagggcagtaacaagtgcatactacagaggagcattaggggcc
atgttagtgtatgacataacaaaaagacaatcatttgatcatgttgctaaatgggttgag
gaactaagatcacattcagataacaccattgtgatcatgctagttggtaacaaaggtgac
cttgttgacttgagagttgttcctactgaagatgcaattgagtttgctgatgaacaagga
ctattcttttatgagacttcagctctcactggtgagaatgttgagactgcatttatcaaa
ttgtttgaagagattaatagggtggtttgtaagaaggcattggaatgtggtaatggttac
tatgatgctgcttctacaattaaaggttcaaagattgatgtaatctcaggtgctgaatta
gaaattactgagatcaagaaattgtcttcttgctcttgttaa

***>RabA-4a***

atggcgagtggaggagggtacggtgatccaaaccagaaaatagactacgttttcaaggta
gttctaatcggtgattctgcggtagggaagtctcaaatacttgctcgatttgcgagaaac
gacttcagcttagactctaaagccacaatcggcgtcgagtttcagactcgcactttggtc
attcaacataagagcgttaaagctcagatctgggacactgctggccaagaacgttataga
gcagttacaagtgcatactacaggggtgctgttggggcaatgttggtttatgacattaca
aaacgtcagagctttgatcacatacctagatggttagaagaactgcgtaaccatgctgat
aagaatatagtcataattcttataggaaacaaaagtgatcttgagaaccagcgagcagta
ccaacagaggatgcaaaagaatttgctgagaaagaaggcttattttcttagagacctct
gcacttgaagcaactaatgttgagacagccttcacgactgttttgacagaaatatttaac
attgtcaataagaagaacctggctgctgatgagaatcaggaaaatggcaactcgacatct
ctagctggcaagaagattattgttcctggtcctgcacaagaaatccctaagaagagcatg
tgttgtcagtaa

***>RabA-4a****

atgggaagtggaggaggaggttatggagatgcaaaccagaggatagattatgtattcaag
gtggttttgatagggggattcagcagtggggaaatcacagatattagctagattttctagg
aacgagttcagtttagattctaagtccacgatcggagttgaatttcaaacacgtacttta
gtcatcgatcataagaccgtcaaggctcagatctgggacactgctggtcaagaacgatac

agagcagttacaagtgcatactacaggggtgctgtaggggcaatgttagtttatgacatc
acaaaacgtcagacctttgatcatataccccgttggctagaagaacttcgtaaccatgct
gacaagaatatagtcatcattcttgcagggaacaagagtgatctcgagaaccagcgtgat
gtacccacagaggatgcaaaagagtttgctgagaaagaaggtttatttttcttagagacg
tcggcattggaagcaactaatgttgaagaagccttcatgacagttttgacagaaatatac
aacattgtcaacaagaagaccctagctgctgatgaaaatcatggaaatggaaactcggca
tctttggctagccagaaacttattattccaggccctgcacaggaaatccctgctaagagc
aacatgtgttgccaggcatcatga

**>RabA-4c**

atggctcagtggcaaggtgattctgatgacgatattgattacatgtttaaggttgtgtta
attggagactctggagttggaaaatctcagcttttaaatcggtttgttagaaatgaattt
cacatgaaatctaaagctacaattggtgttgaatttctgactaagacggttctcatggat
cacaaacatgtcaaggctcagatttgggatactgctggtcaagaaaggtaccaagcaatt
acaactgcatattacagaggtgcaactggtgcattactaacatatgacgtaaccaagcgc
cattcctttaaccatgttgaaaaatggctagacgaactacacatgcatgccgataaaaac
atagttgtcatgctcgttggcaacaagtctgacctgaattctattcgagaagtgccgacc
gaggaagcaaaagactttgccaagcagaaaggtctcttcttcatcgaaacatctgcgctt
gactccaacaatgtagaaccagctttccttggtcttctttcacaaatatatatggcagtc
agtaagaagcacatcactgcagatggacatgaatcaaattgggataaagtaaaccttgaa
tttgtgggaacaaaaaattaaggtctcatcacaagaaccagaatttcaaaaatctaaaagg
agatacaattgttgcagcgttttatag

**>RabA-4d**

atgttgaattgttggaggattgtgacgatgtcgaatttgtacggtgattacaacttgaag
atcgattacgtattcaaggttgtattgattggtgattcagcagttggaaaaactcaacta
ctcgctcgttttgcaaggaacgaatttaacgttgattcgaaggccacaattggggtcgaa
tttcaaaccaaaactttaattattgataataaaactattaaggctcaaatatgggatact
gcaggtcaagaaagatacagggcagttactagtgcttactatcgaggtgcagttggggca
atgttagtttacgacatgacaaagcgtcaatcgttcgatcacatggcaagatggttggaa
gaattgagaggtcacgccgacaaaaacatcgtcatcatgctaattggcaacaaatgtgat
cttgctagtcttagagcagttccaacggaagacgcagaggagtttgcacaaagagagaac
cttttctttatggagacatcagcgctggaatccaccaacgtcgaaacatgctttctaact
attttaaccgagatataccgaatcaatgccaagaaaacactcacttcaaatggtgatgca
gatcatagtgatacttcaggtcttcttaaaggaagtagaataattgttcctggtaatcaa
atggatgctactggaaagaagggtggttgttgttttgcctcatag

**>RabA-4d***

atgtcgaatttgtacggcgattacaatcaaaagatcgattacgttttcaaagttgtatta
attggggactctgctgtcggcaaaacgcaacttctcgcacgattttcaaggaatcaattc
aacgtcgattctaaagccaccatcggcgtcgagtttcagaccaaaactcttataatcgat
aataaaaccgttaaggctcaaatatgggacacggctggtcaagaaaggtacagagcagta
actagtgcgtattatcgcggagcagttggagcaatgttagtttacgacatgacaaagcgt
cagtcatttgatcacatggcaaggtggttagaggaactgaggggtcatgcagacgccaac
attgtcataatgctaattggcaataaatgtgatttgggaactcttagagcagtaccaact
gaagatgcccaggagtttgcacaaagagagaatctattctttatggagacatcagcactt
gagtctactaatgtggaaactgcctttctgactattctaaccgaaatataccgacttatc
agcaagaaaacactcactgccaacgatgacgcagatcctgctggtagttcagggcttctg
aaaggaaccaaaataattgttcctaaccaagatgtgaatgctcttgaaaagaagggtggc
tgctgtggataa

**>RabA-5a**

atggcttcttataacgaagaagagaagaccgaggattaccttttcaaaattgttttaatt
ggtgattcagctgttgggaaatcaaatttacttgcaagatttgccagggatgaattttat
cctaattcaaagtcaactataggagtagagttccaaactcagaaaatggaaattaacgga
aaggaagttaaagcgcagatatgggacacagccgggcaagagaggttcagagctgttacg
tctgcttattataggggtgcagttggagcacttctggtatatgacattagcaggcgccaa
acatttgatagcattggtcgatggctcaacgaacttcacactcactctgatatgaacgtt
gtcacgatacttgttgggaacaagtcagatcttaaggatgcgagggaggtgcctacaacc
gaaggcaaggccttagctgaggcacagggtttgttctttatggagacatcggctctcgat
tcatcgaacgtagtagctgcttttcaaacagttgtaaaagagatctacaacatattaagc
cggaaggttatgatgtctcaagagctcaataaacaggatccttcctggattgaaaatgga
aagactgttgttctacaagaaggggataaagaaaaagaagcagaagctgagacaaaaaag
ggttgttgttcatcttag

## ***>RabA-5b***

atggacgaaaacggtgaaggaggtgaagagtacttgttcaagatcgtgttaatcggcgac
tcagcagtaggaaaatcgaacctactttcacgattcgcacgaaacgaattcgactcgaat
tcaaaagcaacaatcggcgttgaatttcagacgcaaatggtggaaatcgacggtaaagaa
gtgaaggcgcagatctgggatactgctggtcaagaacgtttcagagctgttacttctgct
tactatagaggtgctgttggtgctcttgttgtttatgatattagtaggagaggtactttt
gatagtatcaagaggtggcttgatgaacttactactcaaaatgatagcacggtagcaacc
atgttggtgggaaataagtgtgatttggagaatatcagagaagtgagcacagaggaggga
aaaactcttgcagaagaagaaggtttgttctttatggagacatcagcactcgaatctacc
aatgttcaaacggcttttgagattgttatccgtgaaatttacaataacatcagccgcaaa
gtcttaaactctgattcttataaggctgaattgtctgtgaatcgagtaagcctggttaat
ggggcaggatcaaagaaaaatctgctcaatttttcctgttgttcatga

## ***>RabA-5e***

atgtctagttcagaagatgaaggaggaggagaagagtatctcttcaaagtcgttataatc
ggtgactccgcagttggtaaatcaaatctactctctagatacgctcgtaacgagttcaat
cttcactccaaagctacaatcggtgttgagtttcaaactcagagcttagaaatcgattcc
aaagaagttaaggctcagatttgggatactgccggtcaagaacgcttccgtgctgtaacc
tccgcttattacagaggcgctgtcggtgctcttgttgtttatgatattagccggagaact
acttttgatagcgtcggtcgttggctcgatgaactcaagactcattgcgatacgacggtg
gcaatgatgttggtgggaaacaaatgtgatttggataatataagggcagtgagtgttgaa
gaaggaaaaagccttgcggaatcagaaggtttgtttttcatggaaacttctgctttggac
tctacaaatgtgaaaacagcttttgaaatggttattagagagatatacaacaatgtcagc
agaaaggtcctcaactcagacacttacaaggctgaattatctgtcaacagggttagtctt
gttaacgatggcaactctacatccaagaaaaacaatagctatttttcttgctgttcctga

## ***>RabA-6a***

atggctgatgcatttgatgaggagtgtgattacctcttcaaagctgttttaattggagat
tcaggagttgggaaaacaaatcttatttcaaggtttgcaaaagatgaatttcgacttgat
tccaaaccaaccataggtgtggaatttggttacaagaacatcaaagttagagacaaactc
atcaaagcacaaatatgggacactgctggccaagagaggtttagagctatcacaagctca
tactatagaggagccttaggggcaatgctagtgtatgacataactagaagaacaacattt
attaatatagaaaaatggttatatgagcttagagagtttggaaatgaagacatggtgata
attcttgtagcaaacaaatctgatttgagtcaatcaagacaagttgagaaagaagaagga
aaagggtttgctgaaaaagaaagtttatgtttcatggaaacttctgctttgcataattta
aatgttgaggatgcattcttagaaatgattactaagatacatgatatcataagccaaaaa
agtttggaagaaaaaattaatggccaaagttaagtgttcctaatggaaaggaaattcat
atagttgatgaagtgactgccactaaacaagttaattgttgttcaagatga

***>RabA-6a****

atggatgatgcatttgatgaagagtgtgattacctgttcaaggcagtattgatcggagac
tctggagttggaaaatcaaatctgctttcaagatttgcaaaagatgaattcaggttggat
tcaaaaccaactattggagttgaatttgcttacagaaacatcaaagttagagacaaactc
atcaaagctcaaatatgggacactgctggtcaagaaaggttcagagcaatcacaagctcg
tactatagaggagccttgggagcagtgttggtgtatgacataacaaggcgatcaagttat
gagagtgtaggaaaatggttagtggagctaagggagtttggtggggaagacatggtggtt
attcttgtgggaaacaaatgtgatcttagtgaatcaagagaagttgataaagaagagggt
aaagcgtttgcagaggaagaagggttgtgttttatggaaacctctgctttgaagaatctg
aatgttgaacaagtgtttttacaaatgatcacaaagatacttgagatcacaagtcagaga
agtttggaagctaaaatggatgagaaaccaattagtctttggaatgggaaggagattagt
ttattggccgatgaagtcacagctactaaacaggctccttgttgttcaagatga

***>RabB-1b***

atgtcgtacgattacctcttcaagtacatcatcatcggcgacacaggtgtagggaaatct
tgtctgctccttcagttcaccgacaagaggtttcaacctgttcatgatcttacaattggt
gttgagtttggtgctcgtatgctcaccattgattctaggcctattaagcttcagatatgg
gacactgctggacaagagtcttttagatccatcactagatcttactacagaggagcagca
ggggcacttctagtttatgacattacaaggagagagacatttaatcatttagcaagttgg
ttggaagatgcccggcagcatgcaaatcctaacatgacaatcacgctcatagggaacaag
tgcgatctatctcaccgaagggcggtaagcaaagaggagggagagcagtttgctaaggaa
aatggacttttattcatggaggcttctgccaagacagctcaaaatgtagaagaggctttc
ataaagactgctgcaaagattcttcagaacatccaggatggtgtcttcgatgtgtccaat
gagacatgtggtataaaagttgggtatggacgtccacaaggccagtcaggaggcagagat
ggaactgttgctgcaggaggtgcatgttgcggctga

***>RabB-1c***

atgtcttacgcgtacctcttcaaatacatcatcatcggtgacactggagttggaaagtcg
tgtcttctactacagttcactgacaagcgctttcaacccgtccatgacttgaccattggt
gttgaatttggtgcaaggatgatcactattgataataagccaatcaagttgcaaatatgg
gatacggcgggtcaagaatccttcagatctattacaaggtcgtattacagagggggctgca
ggtgcactgcttgtttatgatataaccaggagggagacatttaatcacttggctagctgg
ttggaagatgcaaggcagcatgcaaatgcaaatatgacaattatgctgattggcaacaaa
tgtgatcttgctcataggcgggctgtaagcaccgaggaaggtgagcagtttgcaaaggag
catgggttgatcttcatggaggcctcagcaaaaactgctcagaacgttgaagaggcattc
ataaaaacagctgcaaccatatacaaaaagattcaagatggacttttgatgtatcaaat
gagtcttatggaataaaagtaggatatggtggaattcctggaccatctggaggtagggat
ggcccttctgctgcaggcagaggctgctgcaattga

***>RabB-1c****

atgcttccctaccttcacaaaaattctcaaaagtcaaaacatctaaaaagaaaatcctac
gcatatattttcaaatacatattaatcggtgacactggagttggaaaatcatgtctccaa
cttcaattcaccgaccaccacttccaacctttccacgacgtaacaattggtgttgaattc
ggcaccaggacaatcaacattgaaaaaattccaataaagttacaattatgggacacagcg
ggtcaagaaaatttagatcaattacaagatcatattatagagatacaacatgtgcatta
ctagtttatgatgtaacaaggagagaaacatttgatcacttggataattggttgaaacaa
atattggaagatggtaatgagaaaatggtagttatgttaatcggaaacaagtgcgatctt
atcgataatcgagttgtgacgaccgaagaaggcgagcaatttgcgagggctaatggtttg
atttttatggagacttcggccaaaactgctaagaatgttgaagaaatatttgtgaaatca
gcttcaactatatgtaataagattaaggatggtgtttttgatgtaaagaatgataattgt
gggattagagtaggatatggtggaggtaggtgtgatgaatttttagatttgggaggaggt

tgctgcaaaacttga

***>RabC-1a***

agtaatttcagaaaaccaaacggttcagcggcgctagcatacgccaagttttctttgact
ttccgcaaccttctactaaatactttacttcttagctttttcttttccttctgtttccac
attccacagaattgattcaacccaagtcttgattttccaaggaaccgaagaaaatatctc
tgagatccaatctaaacaaaacaatatctgaatggatgttacttcctcctcgtcgagtca
cgccgaattcgattacttattcaagcttctgttaattggtgattctggagtcggcaaaag
cacgctgcttctcagattcacctctgataatttcgaggatctttctcctaccatcggtgt
agacttcaaagtgaaatatgttacaattggtggaaaaaagttaaaacttgctatttggga
cacagctggacaagaaaggtttagaacacttaccagttcatattatagaggtgcacaagg
aataattatggtgtacgatgtaacacggcgggaaacttttacaaatctatctgatatatg
ggctaaagaaattgacttatactcaacaaatcaagactgtatcaagatgcttgtaggaaa
caaagttgacaaggaaagtgaaagggttgttagcaaaaaggaagcggtggactttgcaag
gcaatatggatgcctttatactgaatgcagtgcgaaaacccgagtaaatgttgagcagtg
ttttgaagaacttgtagtgaagattttggagacaccaagcctcttggctgagggctcatc
tggtgtgaaaaaaaacatcttcaagcagaaaccacaagagtctgatgcatcaactagtag
ctgttgctcatggtgattatgatgtatttaaagagctatcaccttccacttttcagtatg
atgttaagacatttcattacatgtaccaaaaaactaatttcaatttgttgcgatcaatct
ttaaaatttgcctacttttaatggtctctttcatttta

***>RabC-1c***

atggattcgagttcggggcagcaagaattcgactatttgttcaagttgttaatgattgga
gactctggtgttggaaaaagtagttttcttctcagattcacctccgatgatttcgaagat
ctctcccccaccattggtgttgattttaaggtcaaatatgtcatgatgggggtaaaaaa
ctcaagcttgccatttgggataccgctggtcaggagagatttagaacactcacaagttcg
tactatcgaggagcacaaggaatcatcatggtttatgatgtaacacggagagaaacattt
acaaatctctctgaagtatgggcaaaggaaatagacctttattcaacaaatcaagactgc
gtgaagatgcttgttggaaacaaagtagataaggagggtgatagagttgtgacaaagaaa
gagggaatggacttcgctagggaatatggttgcctctttattgaatgcagtgctaaaact
cgagtaaatgtgcagcaatgctttgaagagcttgttttgaagactcttgatacacctagc
ctcttagctgagggctctaagggagtaaaaaataacattttttaaggacaagccacctcag
tctgatgcatccacaggtggttgttgctgattttaagaagctacttcacttggaagctgc
attttaagaatcacggcctccaagagaaaggcattgcatccatttttcctgggtttctaac
aaatttcctggttactttagccttttagcataagtggaggtcatttttttatttttcaagt
tcctgttttgacttgaatatgtagatgtaatatagtgatgattgttgtctgcaatttctt
gggacttgcgcttaatttcgtgtgaagacgtgcaacttttttatttttaaatcacaatagat
gatccagatcaaattgtatataataataatgtctgagcaacgatatgctccaactgtcga
tgttgcagagcttgagcaataccataaaagcaagcaaacgctgaagataagcatcaaaga
taatcaaataataagatttgaagtta

***>RabC-1b***

atggatttgagtacgagtcaggaattcgattatttgttcaagttgttgatgattggggac
tctggagttggcaagagtagtctcctcctctgtttcacctctgatgctttcgaagatcta
tccccaacaattggtgttgattttaaggtcaaatatgttactatggatggtaaaaagttg
aagcttgccatttgggatacagctggtcaggagagatttagaacactaacaagttcttat
taccgaggtgcgcaagggatcattatggtttacgatgtcactcggcgagatacatttaca
aatctctctgaagtatgggctaaggaaatagacctctattcaacaaatcaggactgcatc
aagatgcttgttggaaacaaagtggataaggagagtgatagagttgtgacaaagaaagag
ggaatagactttgcaagggaatatggttgcctatttactgaatgcagtgctaaaactcga
gttaacgtacaacaatgctttgaagagcttgttttgaaggttttggacacacctagcctc
ttagccgagggatctaaaggggtcaaaaagaacattttttaaggacaagccaccccagtct

gatgcagccacaagtggttgttgctaagaaaaggctatttcactcaaagtaaaaaatcac
tgcctattgaagatctcaaaaaaaagggcgttgaatccgttttcagaatcagagttctaa
aacacgcttattggtgtttatttagcaccagctgaagtcttctaaaaaagaatccatgct
cctggtttgtctggaatatttgtaatataatgattatggttttctcttctttttccttat
ttggattttattattattattatttgcatttaatttcatatattttgacgtgcaagaatc
gtggatatatcatgttatgaaaaccaattctaattgtaaacaatgcctggacaacaatgt
catgtaactttttttaaatagacaaatgttagtgtactagttttgtaagtatttttccttt

***>RabC-2a***

atgcttagaaactataccccatttgcatttcatcattattactacacaacacaacacaaa
ctttacctttttctttctctctctctctctctcttgataggagaatgagttcttcttct
ggtcaaagcagcagtagctatgatctctctttcaagatcttgttgattggtgattctgct
gttggaaaaagcagcttaattcttagcttcatctctgcttctgttgaagatctttccccc
actattggtgttgattttaagatcaagcttctaacagtaggtggcaagagattgaaacta
actatttgggacactgctgggcaggaaaggttcagaacactaactagttcttactataga
ggagcacaaggaatcattctcgtttatgatgtaacaagaagagatacctttacaaactta
tcagaggtgtggtccaaagaagtggaactttattcaactaatcagaattgtgtgaagatg
ctagttggaaataaagttgatagagactctgaaagggttgtgagcacagaagagggttta
gctctagccaaagagtttggatgtttgtttttttgaatgcagtgccaaaactcgagaaaat
gtagacaagtgtttcgaagaacttgcactaaagataatggaagttcctagtcttttggaa
gaaggatcaacatcagtgaaaaggaatattttaaagcaaaaacaggaacctcaagcatca
caagatggtggttgttgctcttaaaatgctgcaatttgaacattgagttttaactaccac
atattgaactggtcctcttatgtaatattcttccatagtttcttattggctccattgtct
tttggttgctcaatagattgtaccacaactaatggggaattggcgataataacgcaaatc
aactggtgtaaatttctatactttctgtcattgtttttagcctatacgtattcaaatcaa
tacaaccttgtgcaa

***>RabD-1***

atgagcaacgaatacgattacctatttaaggttcttataatcggagactcctccgttgga
aagtcttgcttgcttctcagattcgccgatgactcctatgtcgatacctacattagtacc
attggcgtcgatttcaaaatcagaactgtggaattggaagggaaaaccgtcaagctgcag
atttgggatacggctggacaggagcgattcaggactataactagcagttattatagagga
gcacatggaattattattgtttatgatgtcactgacatcgaaagtttcaacaatgttaag
caatggttgcacgaaattgatagatatgcaaatgacactgtatgcaagctacttgtaggg
aacaaatgtgatctttctgataacaaggttgttcacacccacactgccaaggcatttgca
gacgagcttggtatccctttccttgagacaagtgctaaagactcagtcaatgtggagcag
gctttcttgactatggcagcagagattaagaagaagatgggaaaccaaccagctagtagt
aagtcagcggaatctgttcagatgaaggggcaaccaatcccacagaagagcaactgttgt
ggctag

***>RabD-2a***

atgaatcccgagtatgattatctgttcaagcttcttcttattggagactctggtgttggc
aaatcatgccttcttctgagatttgctgatgattcttacattgacagctacataagcacc
attggagttgattttaaaatacgcaccgttgagcaggatgggaagaccattaagctccag
atttgggatactgctgggcaagaacgatttaggacaatcaccagtagctactaccgtgga
gcacatggaatcattattgtttatgatgtgacagatgaagagagcttcaataatgtgaag
caatggctcagtgaaattgaccgctatgccagtgataatgttaacaagctcttggttggc
aacaagtgtgatctgacagcaaatagagctgtgccatatgaaacagctaaagcatttgca
gatgaaataggcatacctttatggagacaagtgcaaaagattctacaaatgtcgaacag
gcctttatggcaatggcttcttccatcaaggatagaatggcaagccaacctacaaacaat
gcaaggcctccaaccgtgcagataagaggacagccagttgggcagaaaggtggctgctgc
tcatcttaa

>RabD-2a*

atgaatcccgagtatgattatctgttcaagctccttcttattggagactctggtgttggt
aaatcatgccttcttctaagatttgctgatgattcatacattgatagctacataagcacc
atcggagttgattttaaaatacgaactgttgagcaggatgggaagacaattaaactacag
atttgggatactgccgggcaagaacgatttaggacaataaccagtagctactatcgtggg
gcacatggaatcattattgtttatgacgtgacagatgaagagagcttcaataatgtgaag
cagtggctcagtgaaatcgaccgctatgccagtgataatgttaacaagcttttggttgga
aacaagagtgatctgacagcaaatagagctgtctcatatgatacagctaaagaatttgca
gatcaaatcggcataccttttatggaaacaagtgcaaaagatgctacaaatgtggaaggg
gcattcatggccatggctgctgccatcaaggatagaatggcaagccaaccgtccgcaaac
aatgcaaggcctccgacggtgcagattagaggacaaccagttgggcaaaaaggtggttgc
tgctcttcttag

>RabD-2c

atgaatcccgaatatgactatttgttcaagcttttgttgattggagattctggtgtgggc
aagtcatgtctcctcctgaggtttgctgatgattcataccttgacagctatatcagtaca
attggagtggactttaaaattcgcactgttgagcaagacgggaagaccattaaacttcaa
atttgggacactgctggtcaagaacgtttccggactatcactagcagctactatcgtggg
gctcatggcataattgttgtttatgatgtcactgaccaagagagctttaacaatgttaag
cagtggctgaatgaaattgaccgttatgcaagtgaaaatgtaaacaagcttctagttgga
aacaagtgtgatctcgcggcaaataaagttgtgtcctctgaaacagcaaaggcatttgca
gatgaaattggaattcctttcatggaaactagtgctaaaaatgccaccaacgtggaacag
gctttcatggccatggctgctgaaattaaaaacagaatggcaagtcaaccagcgaacaat
gccaggcctccaacagtccaaattcgaggacagccaatgaaccaaaagtctggttgctgc
tcgacttag

>RabE-1a

atggctgcaagagctcgttccgattacgattatctcattaagcttctccttatcggtgat
agcggtgtgggtaaaagttgccttctattgcgtttctcagatggatcattcacaactagt
tttataacaaccatcggcattgatttcaaaataaggacaatagagcttgatggaaagcga
atcaaattgcaaatatgggatacagctggtcaagagcgtgttcgaactattacaactgct
tactaccgtggagccatgggcattttgcttgtgtatgatgtcactgatgagtcgtcgttt
aacaacatcaagaattggattcgcaacattgagcaacatgcttccgataatgtcaacaag
atattagtggggaacaaggctgacatggacgaaagcaaaagggctgttccaacttcaaag
ggtcaagctctggcagatgaatatggcatcaagtttttgaaactagtgcaaaaactaat
atgaatgtggatgaggttttcttttcaatagctcgggacatcaaacaaagacttgcagaa
agtgactcaaaggctgagccccagtcactcaagattaaccaatcagaccagggagctggg
tctgctcaagccggacaaacatctgcttgctgtggttcttag

>RabE-1a*

atggctgctcgaccagcaaagcctcgtgccggtgctgatcccgattatctcataaagctt
cttttgatcggagatagtggtgttggaaagagttgccttcttctacgattctccgatggt
tctttcacaactaattttattacaaccttaggcattgattataaaaatagagccattcag
ctggatggaaaaaagatgatgatccaagtttgggatactgcaggtcaggagcggttccga
acaattacaaatacttactatcgcggtgctatgggagtattgctagtctacgatgttact
gacgaattttcatttaacaatatcaaaaattggatgcatagcattgagcaatatgcttcc
gacaatgtcaacaaaatactggttgggaacaaagctgacatgaatgaaaggaaaagggcc
gtgcctacctctaagggccaagcactagccgacgagtatggaatcaaatttttcgaaaca
agtgcaaagacaaatttaaatgtggacaaagttttcttctcgattgtgaaagatataaaa
cgaaggcttgatgacattgactcgaagacaccaccgataggaattagaatcaacagacaa
aatcaggcaaacggagcgtggaatgcacagaagttggcgtgctgtggttaa

**_>RabE-1c_**

atggcagcagcaccggcaagagctcgtgccgattacgattacctcatcaagcttcttctt
atcggcgacagtggtgtggggaagagttgtcttcttttgagattttccgatggttccttc
acaaccagttttatcaccaccattggaattgatttcaagataagaaccattgaacttgat
ggcaaacgcattaagttacaaatctgggatacagctggtcaggagagattccgaactatt
actacagcttattaccgtggagccatgggtatcttgctggtttatgatgttactgatgaa
gcatctttcaacaatattaggaattggattcgcaatattgaacaacatgcttctgacaat
gtaaacaagatacttgtgggaaacaaggctgatatggatgaaagtaaaagggctgtacct
accgccaaaggtcaagctcttgctgatgaatatggtatcaagttctttgagactagtgca
aaaacgaacatgaatgtggaggaagttttcttttcaatagcaagagacatcaagcaaagg
cttgcagacacagattctagagccgagccccagactatcaagattaaccaacaagattcg
gcagccgatggtggtcaagctgcacaaaaatcagcttgctgcggttaa

**_>RabE-1c*_**

atggctgctccaccggctagggctcgtgccgattacgattatctcattaagcttctcttg
atcggcgatagcggtgttggaaaaagttgccttcttttgcgtttttctgatggttccttc
accaccagtttcatcacaactataggcattgattttaagataagaactattgagcttgat
ggcaaacggatcaagctccaaatttgggatactgcagggcaggagcggtttcgaacaatt
acaacagcttactaccgtggtgctatgggcatattgctagtctatgatgttacagatgaa
gcatcatttaacaatatcaggaattggattcgcaacattgagcaacatgcttcggacaat
gttaacaaaatactggtgggaaacaaagcagacatggatgaaagcaaaagggctgtgcca
acatccaaaggccaagcactggctgatgagtatggaatcaaattctttgaaactagtgca
aaaacaaatctaaatgtggaggaagttttcttctccatagcaagagacataaaacaaagg
cttgccgacactgataacaaggcagagccaacaaccatcaaaatcaaccaagactctgca
gtgggggctggacaagctgcgcaaaaatctgcttgctgtggttga

**_>RabF-1_**

atgggttgcggctactcccttccagatagggattcaaggcagctaggtcgacccaattca
gagaatggtggaggtcaagacgctaagaatcttcgcgtcaagcttgtcctcttaggcgat
tctggtgttggtaagagctgtattgttctgagatttgttcgtggtcagtttgacccgaca
tcgaaggtaactgttggagcttctttcttgtcacaaacaatcgctcttcaagactctaca
acagtcaagtttgaaatatgggataccgctggtcaagagaggtatgctgcattggcacca
ctgtattatcgtggtgcagcggttgcagttattgtctacgatataacaagcccagaatct
ttcagcaaagcacagtactgggttaaggagctacaaaaacatggaagccctgatatagtg
atggcattagttggtaataaagctgaccttcacgagaagcgggaagtggctgttcaggat
ggcatggactatgcagagaagaatggaatgtttttcatagagacatctgcaaagacagcg
gataatataaatgaactgtttgaggaaattgccaaaagactgcctcgcccagtaattact
taa

**_>RabF-2b_**

atggcaaggaataagagtttacaagccaaactggtacttctgggggacatgggaactggg
aagacgagtttggttcttagatttgtcaaaggtcaattttcggattaccaggaatcgaca
attggagcagcatttttactcaggttttgtcattaaatgaagcaactgtgaaatttgat
atatgggacacagcagggcaggaacgataccacagtttggctcctatgtat

**_>RabG-3a_**

atgtcattgcgcagacgaaccttgctcaaggtcattgtcctcggcgatagcggggttgga
aaaacctcgttgatgaatcaatatgtgcacaacaagtttagtcaacaatataaggctact
attggtgctgattttgtcactaaagaactccaaattgacgacagactcgtcactctacaa
atatgggacactgctggacaagagagattccaaagtcttggagttgcattttatagaggg

gcggattgctgtgttcttgtgtatgatgtgaacgtcatgaagtcgtttgattccctcgac
aactggcacgaggagtttctcaaacaggcaaaccctcctgatacaaggacatttccgttt
atattgcttggaaacaagattgatattgatggtgggaatagtcgagtggtttctgagaag
aaagcaaaggactggtgtgcttcaaaagggaatattccctactttgagacatctgcaaaa
gaggattacaatgttgatgctgcattcctgtgtattgcaaagactgccctggccaatgag
cgtgaacaggacatatattttcaagggattcccgaggcggttccagagaatgagcagaga
ggtggtggatgtgcatgctga

## >RabG-3b

atgtcattacgcaaacgaaccttgctgaaagtgattgttctaggagacagcggggttgga
aagacttctttgatgaatcaatatgtgcacaacaagtttagtcagcaatataaagctaca
attggtgctgattttgtcactaaagaactgcagatcgatgacagactcgttactctacaa
atatgggacactgcagggcaagagagatttcaaagccttggtgttgcgttttacagagga
gcagattgttgtgttctagtctatgatgttaatgtcatgaagtcatttgatacgcttgag
aactggcacgaggagtttctcaaacaggcaaacccttctgatccaaggactttcccattt
atattgcttggaaacaaaattgatattgatggcgggaatagtcgtgtggtttctgaaaag
aaagcgaaggactggtgtacttcaaaagggaatattccttactttgagacatctgcaaaa
gaggactacaatgttgatgctgcattcctttgtattgccaagactgctttagccaatgag
cgtgaccaggacatatattttcaacctattccagaggctgctccagagaatgagcagagg
ggtggatgtgcatgctga

## >RabG-3b*

atggatatttcacatagaaagagaactttgcttaaggttatcgtccttggagatagtggg
gtggggaaaacgtctttgatgaatcaatatgtttataagaaatttagccaacagtataaa
gccacgattggagctgattttgttacaaaggagatactagttgacgacaaactagtaacc
ttgcaaatttgggatacagcaggacaggaaaggtttcatagtcttggagctgcattttat
agaggggcagattgctgtgttttggtatatgatgtaaatatacacaaaacatttgataca
ttaaacaattggcatgatgatttcttaaacagacagatacagaaaatcctgatgcattt
cccttttgtattactcgggaacaaggttgatgtagatggtggaaacagtagaagggttact
gagaagaaagctagggaatggtgtgcttctaggggtaacataccatattttgagacctct
gcaaaagagggatacaatgttgatgatgcattttatgtgttgctaaagttgcatcacaa
aatgaacgtgatctagatatttatttccgtggaatatctgaaactccttcagaaccagaa
caacaaagtggttgtgcatgctaa

## >RabG-3c

atgcatactgaaaaaatagtgtgcttttgggagttattaagtcttggcgttgctttctac
cgaggtgctgattgttgcgtccttgtttatgacgtgaacgtcatgaaatcttttgaaaac
cttaaccattggagagaagaatttctcattcaggctagtccatctgaccctgaaaacttt
ccttttgttgtcttgggaaacaaaatcgatgttgatggtggaaatagcagagttatttct
gagaagaaagcaaaggcatggtgtgcttcaaagggaaacataccatactttgagacatct
gcaaaagaaggatttaatgttgaagctgctttccagtgtatagcaaaaaatgcactcaag
aatgaacctgaagaagaaatgtatctgcctgacacaatcgatgtcggaagtggtggccga
caacagagatcgactggctgtgaatgttga

## >RabG-3d

atggcttctcgccgccgcatgttattgaaagtcattatcctcggagatagcggggttggc
aaaacatcactcatgaatcagtatgtgaatagaaagtttagtaatcagtataaggctacc
attggcgctgatttccttaccaaggaagttcaatttgaagataggttgttcacattgcag
atctgggatactgctggtcaggagaggtttcaaagtcttggcgttgctttctaccgaggt
gcagactgctgtgtccttgtttatgatgtaaatgtcatgaaatcttttgaaaaccttaac
cactggcgagaagaattcctcattcaggccagtccatctgaccctgaaaacttcccattt
gtggtgttgggaaacaaaatagatgttgatggtgggaatagccgagttgtttctgagaaa

aaagcaaaagcatggtgtgcttccaaggggaacataccgtactttgagacctctgcaaag
gagggatttaatgttgaagctgcttttgagtgtataaccaaaaatgctctcaagaatgaa
cctgaagaagaattatacatgcctgaagcaattgatgttggcggtggtggacagcaacag
aggtcaacagggtgtgaatgttga

***>RabG-3e***

atggcttctcgccgccgcatgttgttaaaagttataatcctcggtgatagtggggttggt
aaaacatcattgatgaatcagtatgtgaatcgtaagtttagtaatcagtacaaggctacc
attggtgccgatttcctcaccaaggaagttcaatttgaagacaggttgttcacattgcag
atttgggatactgctggnnnnnnnnnnnnnnnnnnnnagcggtttcaaagtcttggtgtg
gctttctaccgaggtgctgattgttgcgtccttgtttatgacgtgaacgtcatgaaatct
tttgaaaaccttaaccattggagagaagaatttctcattcaggctagtccatctgaccct
gaaaactttccttttgttgtcttgggaaacaaaatcgatgttgatggtggaaatagcaga
gttatttctgagaagaaagcaaaggcatggtgtgcttcaaagggaaacataccatacttt
gagacatctgcaaaagaaggatttaatgttgaagctgctttccagtgtatagcaaaaaat
gcactcaagaatgaacctgaagaagaaatgtatctgcctgacacaatcgatgtcggaagt
ggtggccgacaacagagatcgactggctgtgaatgttga

***>RabG-3f***

atgccgtcccgaagaagaactctattgaaggtcatcattctcggtgacagcggggtgggg
aagacatctttgatgaatcaatatgtgaataagaagtttagcaatcagtataaggcaacc
attggagcggatttcttaaccaaagaagtgcaatttgaagataggcttttcactttacag
atttgggatacagctggccaggaaaggttccaaagcctaggagttgctttctatcgcggt
gctgattgctgtgttcttgtatatgatgttaattcaatgaaatcatttgaaaaccttaac
aactggagggaggaatttctgattcaagcaaatccatccgatccagagaattttcctttt
gttgttataggaaacaagatagatattgacggtgggaacagtagagtggtctcagaaaag
aaggcccgggcatggtgtgcatcaaaaggaaatatcccatattatgagacatctgccaaa
gagggtattaatgttgaagaagcattccaatgcatagcaaagaatgccctgaaaagtggg
gaagaggaagaactatacctgccagacacaattgatgttggaaacagcaatcaacaacgg
gcaacaggatgtgagtgctga

***>RabH-1d***

atggcgccagtttcagctcttgctaagtacaaattggttttcttaggcgatcaatccgtc
ggtaaaaccagcatcatcactcgcttcatgtacgacaaattcgataacacctatcaggct
acaatcggcattgattttctatcaaaaaccatgtatcttgaagatcgaactgttcgactg
cagttatgggatacagctggacaggagagatttagaagtctaattccaagctacattagg
gactcatctgttgctgtcattgtgtatgatgttgcaagtcgtcagacttttctaaacaca
tcaaagtggattgaagaggtgcgcagtgagagaggaagtgatgttattgttgttcttgtt
gggaacaaaactgaccttgtggataagaggcaagtctccacagaagaaggggaagcgaag
tctcgggagctaaacgttatgtttattgaagctagtgccaaagccggctttaatataaaa
gctctctttcgaaaaattgctgctgcattacctggaatggaaacactatctacaacaaaa
caagaagacatggtcgatgtgaacctgaggtcttctgccagccatgactctcaacctcag
tctggtggttgttcttgctga

***>RabH-1d****

atggcgccagtgtcagctcttgcaaagtacaagctggtttttttaggtgatcaatctgtg
ggtaaaaccagcatcattactcgcttcatgtatgataaatttgacaacacttatcaggct
acaattggtattgattttctgtcgaaaactatgtatcttgaagatcgaactgttcggctg
cagttgtgggatacagctgggcaggaaagatttagaagtcttattccaagctacatcagg
gactcgtctgttgctgtcatagtttatgatgttgcaagccgtcaaactttccttaacacg
tcaaagtggattgaagaggttcgcagtgagagaggcagtgatgttattgttgtgcttgtt
gggaacaaaactgaccttgttgaaaagaggcaagtctctacagaggaaggggaagctaaa

tctcgtgagcttaatgtcatgttcattgaagctagtgcaaaagccggctttaatataaag
gctctctttcgaaaaattgctgctgcattacctggaatggaaaccctatcttccacaaaa
caagaagatctggttgatgtgaacctaaaatcttctggtggccatgattctcaaactcag
tctggtggatgttcttgctga

**>RabH-1e**

atggcaaccgtttcacctctcgccaaatacaaactcgttttcttaggtgatcaatcggtt
ggcaagaccagcatcatcactcgtttcatgtatgacaaattcgatactacctatcaggcc
actattggtattgatttttgtcaaaaacaatgtaccttgaagatcgaacagttcgattg
cagctttgggataccgcaggacaagaaagatttagaagtctgattcctagctatatccga
gattcttcggtcgcagttattgtatatgatgtagccaaccggcaatcatttcttaacact
aacaggtgggttgaggaagtacgcacagaacgaggcagcgatgttgttatcgttttggtt
ggaaacaaaactgatcttgttgaaaaaaggcaagtctctatagaggaaggagatgccaag
tcgcgtgagagcggtatcatgtttatagaaaccagtgcaaaagcaggatttaatatcaag
cctttatttcgcaagattgctgcagctttgcccgggatggaaactctttcttcaacaaaa
caagaagacatggttgatgtaaatttaaaatccaccacgaattcatcccagacagagcag
caaggtggaggttgctcatgctaa

**>RabH-1e***

atggcgacggtttcccctctcgccaaatacaagctcgttttcttaggcgatcaatcggtt
ggtaaaaccagcatcatcacccgtttcatgtacgacaaattcgacacaacctatcaggct
actattggtatcgatttttgtcaaaaacaatgtaccttgaagatagaactgttcgtctg
cagctttgggatactgcaggccaagaaagatttagaagtcttattccaagctacataaga
gattcttctgttgcagttattgtatatgatgtagctaacaggcaatcatttctgaacact
aacaagtgggttgaggaggtacgtcaagaacgtggcagtgatgttatcattgtattggtt
ggaaacaaaactgatcttgtcgataaaaggcaagtttctatagaggaaggagatgccaag
tcccgagagtttggaatcatgtttatagaaaccagtgcaaaagcaggcttcaatatcaag
cctttgtttcgtaagattgcttctgccctgccagggatggaaactctttcttccacaaag
caggaagacatggttgacgtaaatttaaaacctacggtgaattcatcccagacagagcag
cagggaggaggttgctcatgttag

**Figure S4.** Relative transcript levels of each *CaRab* gene used in the quantitative RT-PCR analysis, in each of the six chickpea accessions, grown under control conditions. Data are averages of the three time-points sampled during the salt experiment, but for control groups only. Bars represent means relative to transcript levels of two reference genes, *CaHsp90*, Heat shock protein (GR406804) and *CaEf1α*, Elongation factor 1-alpha (AJ004960). Different letters indicate significant differences between data groups based on two-way ANOVA with post-hoc Tukey test (n = 16 ± S.E.M.).

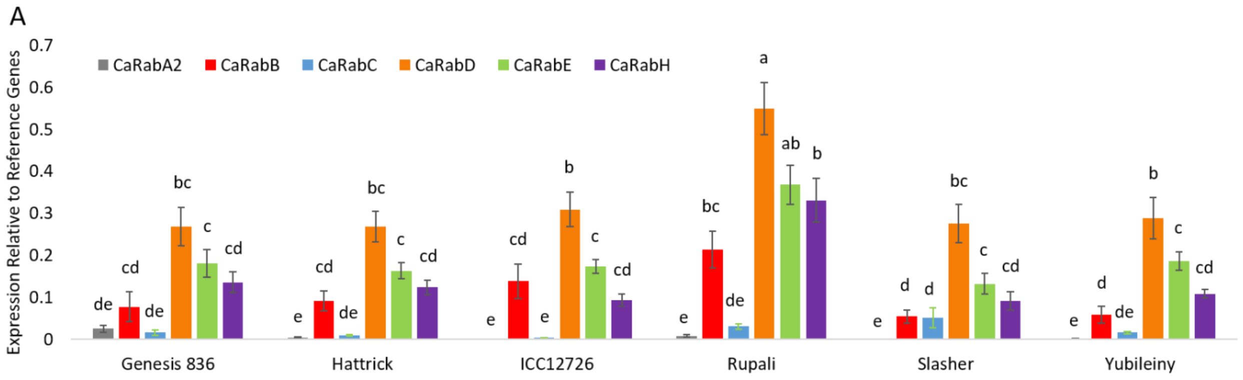

Supplement: Supplementary file 1 — Additional file 1: Table S1. Sequences and information about primers used in the study. Table S2. Full list of chickpea Rab clades and their constituent genes, targeted by qRT-PCR. Including protein and gene IDs and calculated primer efficiencies for each primer set, based on nomenclature presented in [36]. Figure S1. Images of growing chickpea plants in non-stressed Controls and after 9 days since first time of salt application (90 mM NaCl) based on 80% of field capacity moisture. Figure S2. Images of growing chickpea plants in non-stressed Controls and after 1 month since first time of salt application (90 mM NaCl) based on 80% of field capacity moisture. Figure S3. Alignment results, primers design for CaRab genes and sequences of the CaRab gene accessions. Figure S4. Relative transcript levels of each CaRab gene used in the quantitative RT-PCR analysis, in each of the six chickpea accessions, grown under control conditions. [file 12870_2020_2331_MOESM1_ESM.pdf]
